# Secreted phospholipase PLA2G5 acts as a hemolytic factor in sepsis

**DOI:** 10.1172/JCI195001

**Published:** 2026-05-01

**Authors:** Michihiro Takahama, Krysta S. Wolfe, Gabriella Richey, Madison Plaster, Anna Czapar, Fabian Hernandez, Denis Cipurko, Tatsuki Ueda, Yoshimi Miki, Yuki Nagasaki, Yoshitaka Taketomi, Tatsuya Saitoh, Tadafumi Kawamoto, Steven M. Dudek, Makoto Murakami, Nicolas Chevrier

**Affiliations:** 1 Pritzker School of Molecular Engineering, The University of Chicago, Chicago, Illinois, USA.; 2 Laboratory of Bioresponse Regulation, Graduate School of Pharmaceutical Sciences, The University of Osaka, Osaka, Japan.; 3 Pulmonary and Critical Care, and; 4 Section of Infectious Diseases and Global Health, Department of Medicine, The University of Chicago Medical Center, Chicago, Illinois, USA.; 5 Lipid Metabolism Project, Tokyo Metropolitan Institute of Medical Science, Tokyo, Japan.; 6 Laboratory of Microenvironmental and Metabolic Health Sciences, Center for Disease Biology and Integrative Medicine, Graduate School of Medicine, The University of Tokyo, Tokyo, Japan.; 7 Global Center for Medical Engineering and Informatics, The University of Osaka, Osaka, Japan.; 8 School of Dental Medicine, Tsurumi University, Yokohama, Japan.; 9 Division of Pulmonary, Critical Care, Sleep and Allergy, Department of Medicine, University of Illinois at Chicago, Chicago, Illinois, USA.; 10 AMED-CREST, Japan Agency for Medical Research and Development, Tokyo, Japan.

**Keywords:** Infectious disease, Inflammation, Bacterial infections, Biomarkers, Expression profiling

## Abstract

Sepsis is a systemic response to infection with life-threatening consequences such as hemolysis, a predictor of mortality risks for the disease. Here, by measuring organism-wide changes in gene expression, we discovered that the secreted phospholipase PLA2G5 is induced in colon cell types during sepsis. The genetic deletion of *Pla2g5* and treatment with a PLA2G5 antibody were both associated with protection from lethal sepsis. Treatment with a PLA2G5 antibody during sepsis was associated with increased splenic red pulp macrophages and improved iron homeostasis, linking PLA2G5 to red blood cell homeostasis during sepsis. Mechanistically, bloodborne PLA2G5 led to intravascular hemolysis through its lipolytic activity on red blood cell membranes. In humans with sepsis due to bacterial, fungal, or viral infections, the serum level of PLA2G5 was elevated and predictive of disease severity and mortality. We conclude that sepsis corrupts PLA2G5 into becoming an intravascular hemolytic factor which is toxic for host red blood cells.

## Abstract 2

Bloodborne PLA2G5 damages red blood cells during sepsis. Its blood levels predict disease severity and mortality in humans, and blocking it improves survival in mice.

## Introduction

Sepsis is a systemic response to infection with life-threatening consequences for the host (1). While many molecular and cellular factors have been linked to the damaging effects of sepsis on the body, our knowledge of the molecular mechanisms underlying these harmful effects remains incomplete (2–4). For example, intravascular hemolysis is a severe, well-established complication of sepsis and other disorders such as sickle cell disease, malaria infection, or β-thalassemia (5). In homeostatic conditions, damaged red blood cells are removed from the circulation through phagocytosis by macrophages in the spleen and liver. However, in disease conditions such as sepsis, intravascular hemolysis leads to the destruction of red blood cells and the release of free hemoglobin, heme, and iron into the circulation (6). These degradation products lead to toxic effects for the vasculature and tissues through, for example, oxidative stress (7) and the amplification of damaging inflammatory signals (8, 9). In addition, the plasma levels of free hemoglobin, heme, and iron have each been linked with increased mortality in sepsis (10–12). However, the molecular mechanisms underlying hemolysis during sepsis remain unclear, with candidate mechanisms including the coagulation and complement systems or the direct membrane effects of lipopolysaccharide (13). As a result, we lack suitable targets to decrease hemolysis in sepsis.

Secreted phospholipase A_2_ (sPLA_2_) enzymes are found in mammalian tissues and snake or bee venoms. sPLA_2_ enzymes hydrolyze the glycerol backbone of phospholipids to release fatty acids, and lysophospholipids and have been collectively involved in a vast array of biological functions in health and disease (14, 15). Each sPLA_2_ enzyme’s pleiotropic functions result from both its intrinsic catalytic properties and substrate preferences and its changes in expression across cell types, tissues, and disease conditions (14). For example, sPLA_2_ group V (PLA2G5) has several local pathogenic and protective roles linked to various cell types, including macrophages, adipocytes, endothelial cells, bronchial epithelial cells, and cardiomyocytes (16–22). PLA2G5 has also been shown to be induced by lipopolysaccharide (LPS) in a model of acute lung injury (23). However, the expression and function of PLA2G5 during systemic inflammation such as sepsis has not been studied in mice or humans to date.

Here, using organism-wide, spatiotemporal gene expression profiling, we discovered that PLA2G5 is induced in the gut in mouse models of endotoxemia and sepsis. We found that mice treated with a PLA2G5 antibody or lacking the *Pla2g5* gene were protected from lethal sepsis. While PLA2G5 antibody treatment did not impact the levels of inflammatory cytokines or lipid metabolites, it was associated with an increase in splenic red pulp macrophages and improved iron homeostasis. Mechanistically, we found that the lipolytic activity of PLA2G5 led to intravascular hemolysis and multi-organ injury through the degradation products of red blood cells and cell-free hemoglobin, such as heme. Moreover, in humans with bacterial, fungal, or viral sepsis, we found that the serum level of PLA2G5 was elevated and predictive of disease severity and mortality. Taken together, we propose that PLA2G5 is a key factor mediating intravascular hemolysis during sepsis.

## Results

### PLA2G5 is induced in the gut in mouse models of sepsis.

**Figure 1:**
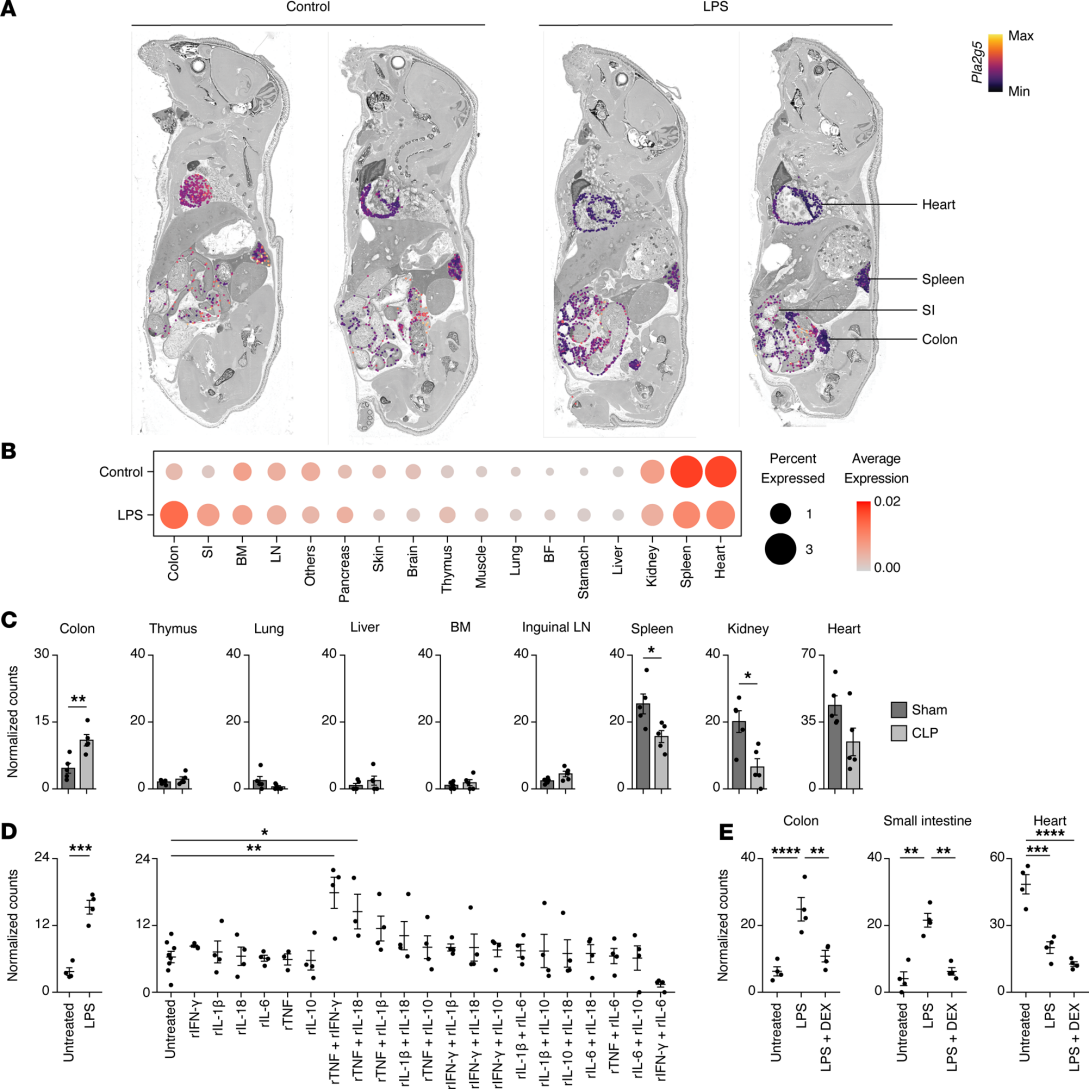
PLA2G5 is induced in the colon during sepsis. (**A**) Whole-mouse spatial transcriptomics (ST) analysis of *Pla2g5* mRNA levels overlaid on a greyscale H&E staining. Shown are whole-mount sections and ST data from 5-week-old mice injected with a sublethal dose of LPS (5 mg/kg) or left untreated as control. The spatial transcriptomics sequencing data and H&E-stained images shown in this figure were reused from a previously reported dataset (GSE224146) and include samples that were originally presented in our previous study (25). SI, small intestine. (**B**) Average expression of *Pla2g5* across all ST array spots covering indicated tissues sampled in sections from **A**. SI, small intestine; BM, bone marrow; LN, lymph node; BF, brown fat. (**C**) Normalized counts for *Pla2g5* from indicated organs at 12 hours post-surgery of cecal ligation and puncture (CLP) sepsis. Means ± SEM are shown (*n* = 5). (**D** and **E**) Normalized counts for *Pla2g5* in the colon (**D**) or indicated tissues (**E**) from mice injected with indicated recombinant cytokines (**D**), LPS with or without dexamethasone (DEX) pre-treatment (**E**), or left untreated as control (**D** and **E**). Means ± SEM are shown (*n* = 3–8). FDR-adjusted *P*-values (limma): *P < 0.05, **P < 0.01, ***P < 0.001, ****P < 0.0001.

We asked if PLA2G5 was transcriptionally regulated across the body during sepsis. Using intraperitoneal injection of a sublethal dose (5 mg/kg) of lipopolysaccharide (LPS) to mimic systemic inflammation (24), we measured changes in *Pla2g5* gene expression across whole-mount, sagittal mouse sections using a custom, large-format spatial transcriptomics method. We chose the 12-hour time point based on our previous organism-wide transcriptomic study and our phenotypic observations (25), as our transcriptomic analysis showed that the largest changes in tissue gene expression occurred at this time. Out of the 17 tissue types profiled using whole mouse sections from animals injected with LPS or left untreated as controls, we found *Pla2g5* transcripts to be significantly upregulated in the colon and small intestine and downregulated in the heart and spleen at 12 hours after LPS injection (Figure 1, A and B). Using whole-tissue mRNA profiling at 0.25, 0.5, 1, 2, 3, and 5 days after sublethal LPS injection from our recent work (25), we observed that *Pla2g5* was strongly upregulated in the colon and small intestine within 6 to 12 hours after LPS injection and returned to baseline within 2 to 3 days (Supplemental Figure 1, A and B; supplemental material available online with this article; https://doi.org/10.1172/JCI195001DS1). In addition, heart and spleen *Pla2g5* mRNA levels were downregulated upon LPS injection and returned to control levels by day 5 after LPS injection (Supplemental Figure 1A). In steady-state conditions, heart and spleen were the 2 tissues with the highest levels of *Pla2g5* gene expression out of 17 tissue types profiled using both whole-mouse spatial transcriptomics (Figure 1B) and whole-tissue RNA-seq (Supplemental Figure 1, A and C). Next, we sought to test if *Pla2g5* gene expression in tissues was impacted after cecal ligation and puncture (CLP), which is considered the gold standard model for sepsis due to its high clinical relevance (26, 27). We used CLP to trigger a polymicrobial infection starting in the abdominal cavity (see Methods) (26). We found that the level of *Pla2g5* mRNA was upregulated in the colon following CLP sepsis (Figure 1C), similar to what we observed in endotoxemia.

Next, we asked what molecular factors regulate *Pla2g5* gene expression in the colon during sepsis. We used gene expression data from the colon of mice treated with 6 recombinant cytokines key to sepsis, IFN-γ, IL-1β, IL-10, IL-6, IL-18, and TNF, by intravenous injection with single or all 15 pairwise cytokine combinations, as reported in our recent work (25). We found that *Pla2g5* gene expression in the colon was significantly upregulated upon injection of recombinant TNF plus IFN-γ or IL-18 (FDR < 0.05), but not in the context of the other cytokine singles and pairs tested (Figure 1D). To corroborate our finding on the induction of *Pla2g5* expression by inflammatory cytokines, we used a corticosteroid drug, dexamethasone, with known antiinflammatory effects to decrease inflammatory cytokine production during systemic inflammation. We found that dexamethasone treatment in our endotoxemia model abrogated *Pla2g5* gene induction in the gut, but did not impact *Pla2g5* downregulation in the heart, suggesting the existence of different signals regulating *Pla2g5* across tissues during sepsis (Figure 1E).

Lastly, to identify the cell types in which *Pla2g5* mRNAs were upregulated in the gut, we used a deconvolution method (28) with publicly available mouse tissue single-cell RNA-seq data (29) as a reference to estimate the cell-type composition for each spot of the arrays used for spatial transcriptomics profiling. Our analysis identified colon goblet cells and secretory cells as the cell types in which the expression of the *Pla2g5* gene is upregulated during LPS-induced systemic inflammation (Supplemental Figure 2, A–C). In addition, using publicly available single-cell RNA-seq data, we found that the *Pla2g5* gene was expressed at steady state in colon goblet cells and secretory cells (Supplemental Figure 3, A and B), albeit at lower levels than during systemic inflammation.

Taken together, our results uncovered that *Pla2g5* is induced in the gut by inflammatory cytokines during sepsis.

### PLA2G5 is harmful for the host during sepsis.

**Figure 2:**
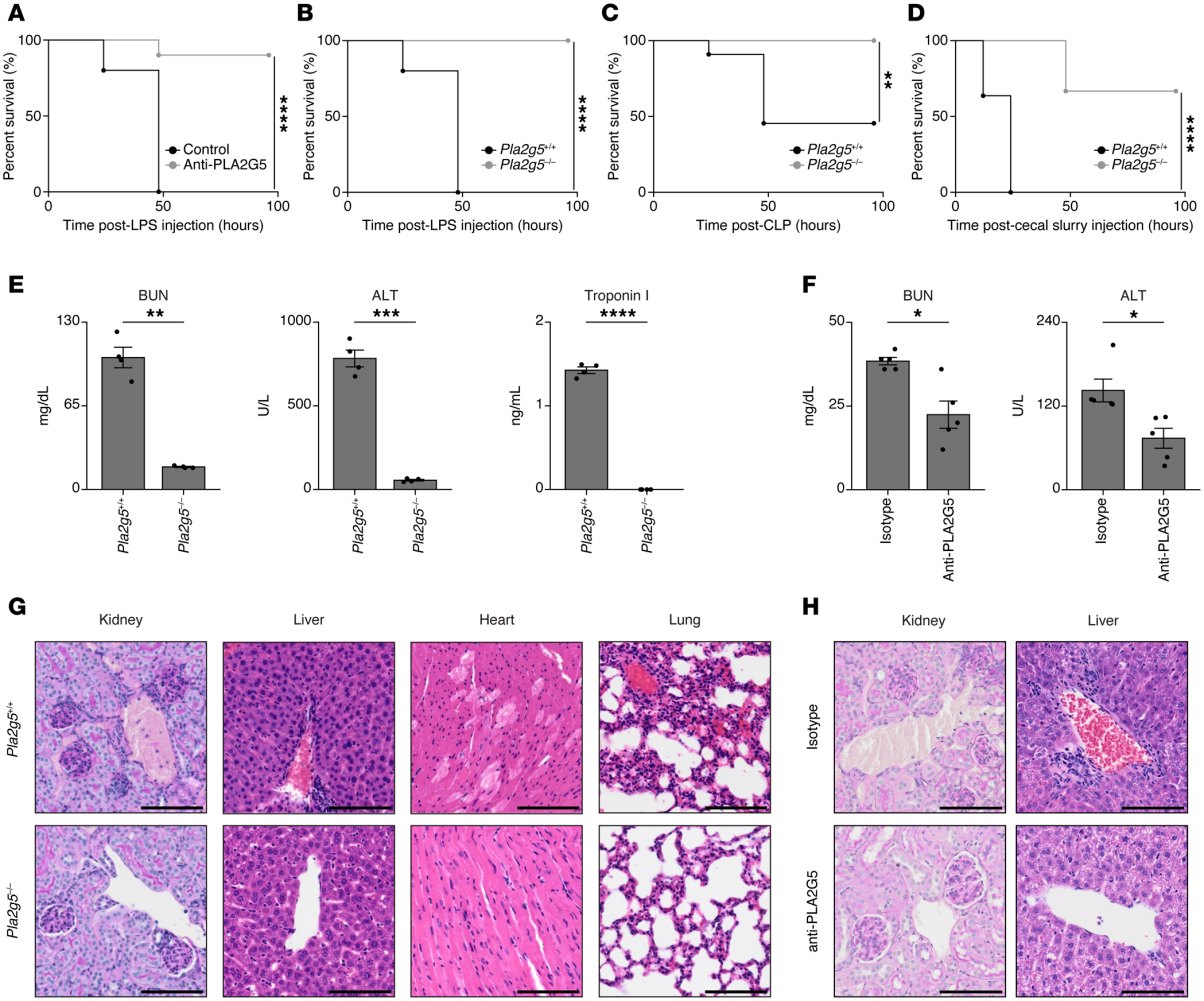
PLA2G5 leads to multiorgan failure during sepsis. (**A**–**D**) Survival curves of mice upon LPS-induced endotoxemia (**A** and **B**), CLP sepsis (**C**) or cecal slurry-induced sepsis using PLA2G5 or mouse IgG1 isotype control antibodies (Isotype) (**A**) or indicated mouse genotypes (**B**–**D**) (*n* = 10–12). (**E** and **F**) Serum levels of indicated biomarkers at 24 hours after injection of LPS in indicated mouse genotypes (**E**) or in the presence of PLA2G5 or mouse IgG1 isotype control antibodies (Isotype) (**F**). Means ± SEM are shown (*n* = 4–5). (**G**–**H**) Histological analysis of kidney (PAS staining) and liver, heart, and lungs (H&E staining) at 24 hours after LPS injection from *Pla2g5*^+/+^ (top) or *Pla2g5*^–/–^ (bottom) mice (**G**) or mice treated with PLA2G5 or mouse IgG1 isotype control antibodies (**H**). Scale bars: 100 μm. Data are representative of 3 independent experiments. *P < 0.05; **P < 0.01; ***P < 0.001; ****P < 0.0001 by log-rank test (**A**–**D**) or 2-tailed Student’s *t* test (**E** and **F**).

To test if PLA2G5 plays a role in the pathogenesis of systemic inflammation, we used a commercially available antibody raised against human PLA2G5. Structural comparison revealed a high degree of similarity between human and mouse PLA2G5 (Supplemental Figure 4A). To quantify this similarity, we used the DALI server, a computational tool that compares 3D protein structures and provides metrics of alignment (30). The DALI analysis provides 2 key values: the Z-score, which reflects the statistical significance of the structural alignment, with higher values indicating stronger similarity, and the root-mean-square deviation (RMSD), which measures the average distance between corresponding atoms in the 2 structures, with lower values indicating closer alignment. Using this approach, we obtained a Z-score of 24.3 and an RMSD of 0.5, confirming that human and mouse PLA2G5 share highly similar 3D structures. Based on the observed structural similarity between human and mouse PLA2G5, we examined whether the antibody raised against human PLA2G5 could interact with mouse PLA2G5. Using immunoprecipitation and Western blotting, we detected binding of the antibody to mouse PLA2G5 (Supplemental Figure 4B). We next investigated whether PLA2G5 antibody treatment improves survival and preservation of body temperature following LPS-induced systemic inflammation. We injected mice with PLA2G5 or isotype control antibodies 1 hour prior to a challenge with a lethal dose (15 mg/kg) of LPS. We found that PLA2G5 antibody treatment significantly increased survival and prevented losses in body temperature upon lethal LPS challenge (Figure 2A and Supplemental Figure 5A). To determine whether the in vivo effects observed with PLA2G5 antibody treatment were attributable to inhibition of PLA2G5 rather than off-target effects of the antibody, we used mice carrying a genetic deletion of the *Pla2g5* gene (31). Importantly, the phenotypes observed in *Pla2g5*-deficient mice recapitulated those observed following PLA2G5 antibody treatment, supporting a role for PLA2G5 in endotoxemia (Figure 2B and Supplemental Figure 5B). We next employed both CLP and cecal slurry models of polymicrobial sepsis to capture the variability and complexity of host responses, as these can lead to distinct transcriptomic profiles (32). In these models, *Pla2g5* deficiency led to increased protection in CLP-induced sepsis (Figure 2C and Supplemental Figure 5C) as well as in cecal slurry-induced sepsis (Figure 2D and Supplemental Figure 5D).

To investigate how PLA2G5 promotes the lethal effects of systemic inflammation, we first measured blood biomarkers associated with organ function and injury. We found that both the genetic deletion of *Pla2g5* and treatment with the PLA2G5 antibody led to a decrease in the serum levels of renal (blood urea nitrogen; BUN), liver (alanine aminotransferase; ALT), and cardiac (troponin I) biomarkers during endotoxemia (Figure 2, E and F). We did not observe differences in other systemic metabolic parameters such as blood glucose, triglyceride, and cholesterol levels, following PLA2G5 antibody treatment during endotoxemia (Supplemental Figure 6). In addition, both the genetic deletion of *Pla2g5* and PLA2G5 antibody treatment were associated with reduced histopathological changes in tissue sections during endotoxemia (Figure 2, G and H). Thus, our results suggested that PLA2G5 regulates tissue damage and survival across several mouse models of sepsis.

### PLA2G5 does not impact systemic cytokine signaling or colon lipid metabolites in sepsis.

**Figure 3:**
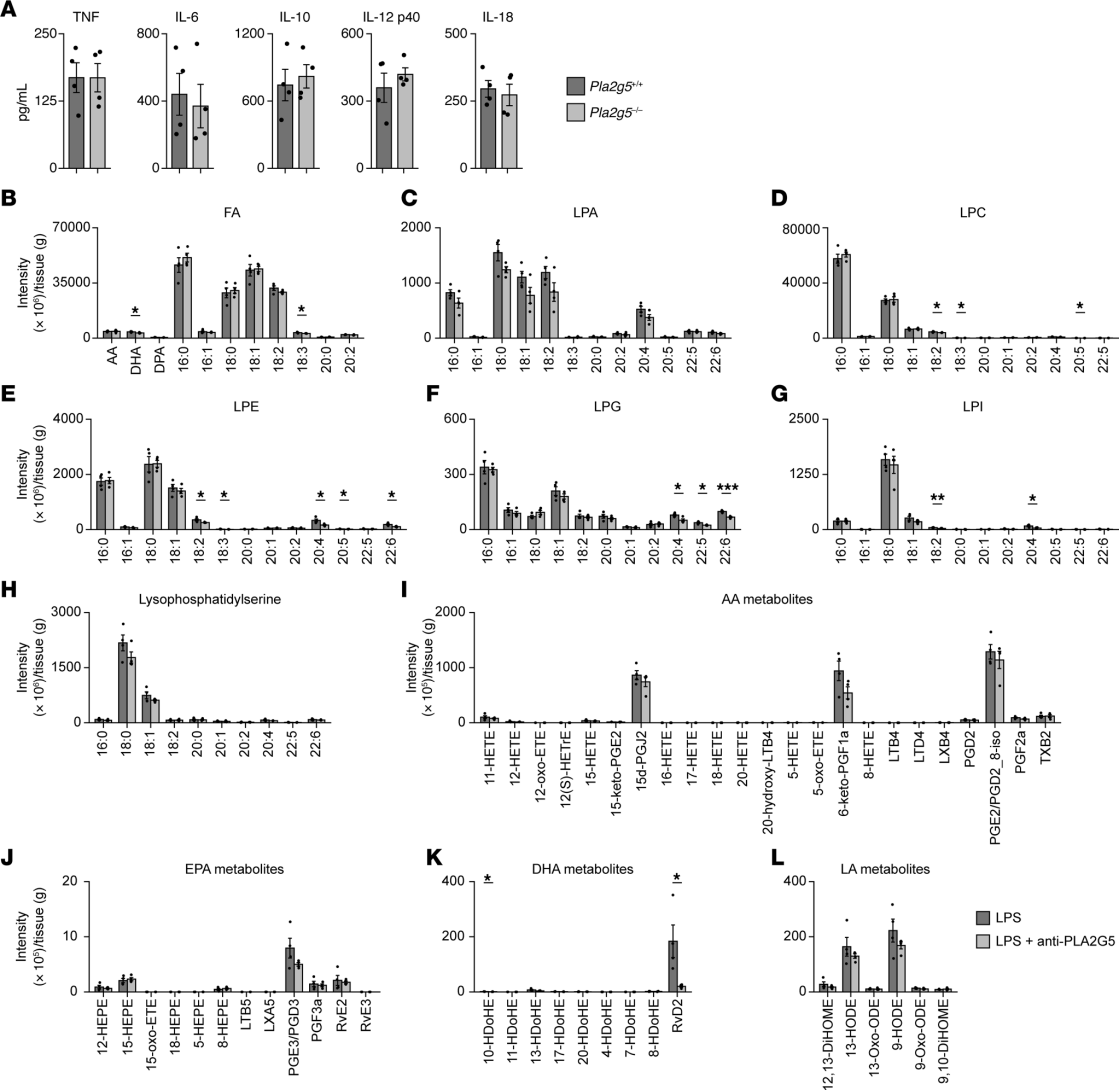
PLA2G5 does not impact cytokine and lipid metabolite levels in the colon during systemic inflammation. (**A**) Serum levels of indicated cytokines in *Pla2g5*^+/+^ or *Pla2g5*^–/–^ mice at 24 hours after LPS injection. Data are shown as means ± SEM (*n* = 4) and representative of 2 independent experiments. (**B**–**L**) Lipidomics profiling in colon tissues from mice after 12 hours after sublethal LPS injection and pretreated with the PLA2G5 antibody or left untreated. Shown are mass spectrometry intensities for indicated lipid metabolites. FA, fatty acid; LPA, lysophosphatidic acid; LPC, lysophosphatidylcholine; LPE, lysophosphatidylethanolamine; LPG, lysophosphatidylglycerol; LPI, lysophosphatidylinositol; AA, arachidonic acid; DPA, docosapentaenoic acid; EPA, eicosapentaenoic acid; DHA, docosahexaenoic acid; LA, linoleic acid; PGE2/PGD2_8-iso, PGE2/PGD2_8-iso-PGA2. Means ± SEM are shown (*n* = 4). *P < 0.05; **P < 0.01; ***P < 0.001 by 2-tailed Student’s *t* test.

We asked if PLA2G5 had detrimental effects on the host during systemic inflammation by modulating systemic inflammatory cytokines, which are common triggers for tissue injury in sepsis (4, 33, 34). We measured the serum levels of TNF, IL-6, IL-12, IL-18, and IL-10 during endotoxemia and found no difference between PLA2G5-deficient and WT mice (Figure 3A). These results suggested that the negative effects of PLA2G5 on the host during sepsis, including multiorgan damage, were independent of cytokines. Next, we hypothesized that PLA2G5 could generate lipid mediators responsible for the negative impact of the secreted phospholipase on the host during systemic inflammation. Using lipidomics on colon tissues, we found that the levels of all the lipid metabolites tested showed little to no changes upon PLA2G5 antibody treatment except for resolvin D2 (RvD2), which was downregulated in LPS-treated mice upon PLA2G5 antibody treatment (Figure 3, B–L). Therefore, the role of PLA2G5 in sepsis appeared to be independent of systemic cytokine dysregulation or the lipolytic activities of the enzyme in colon tissue where it is induced in systemic inflammation.

### PLA2G5 antibody treatment increases splenic red pulp macrophages and iron homeostasis.

**Figure 4:**
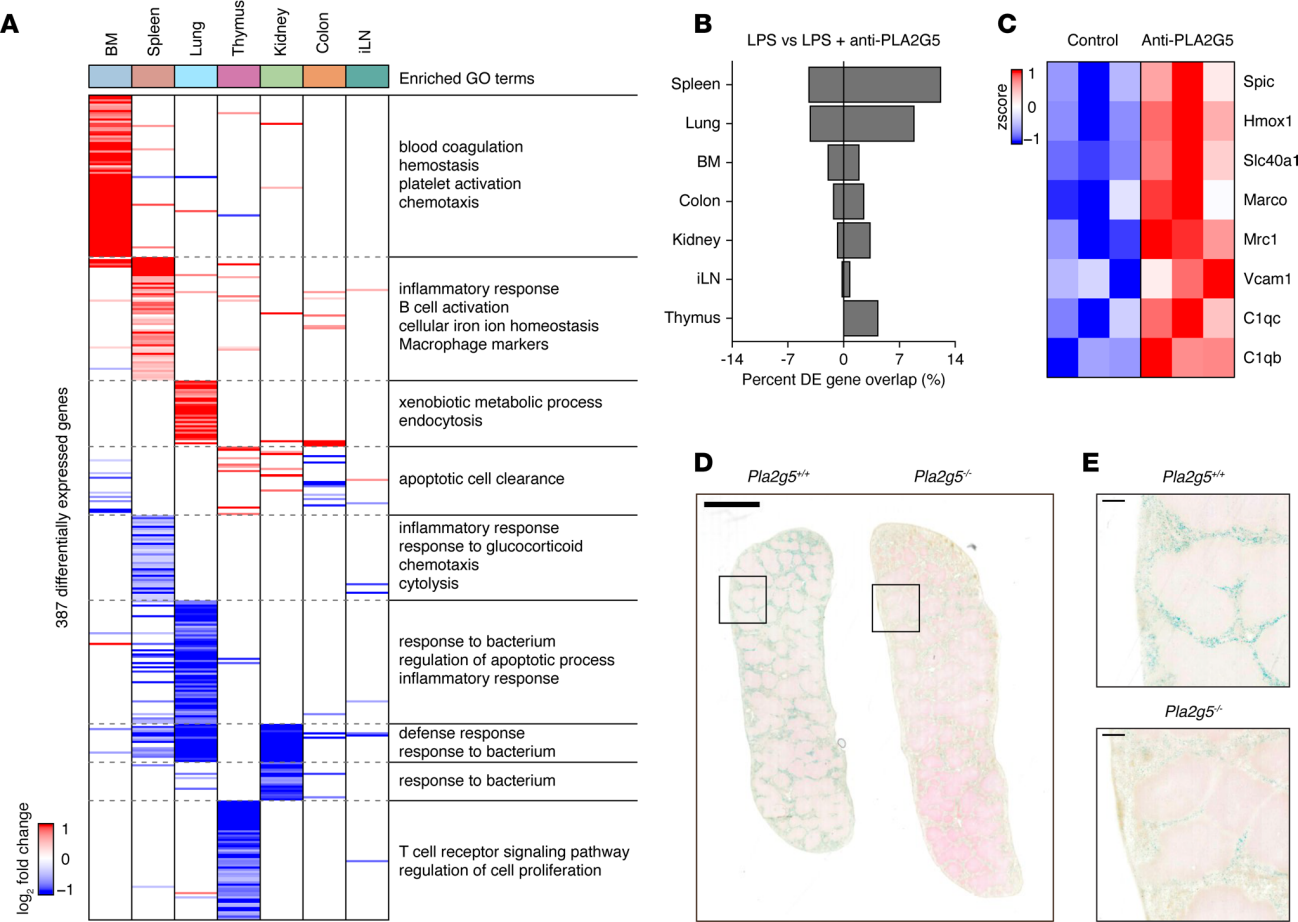
PLA2G5 antibody treatment increases splenic red pulp macrophages and iron homeostasis. (**A**) Heatmap of differentially expressed genes (rows) from whole-tissue mRNA profiles ordered by k-means clustering (dashed lines) and organ types (top, colors) at 12 hours after sublethal LPS injection and PLA2G5 antibody pretreatment. Values are log_2_ fold changes relative to matching tissues from LPS-treated mice without PLA2G5 antibody treatment (FDR-adjusted *P* value < 0.01; *n* = 3–4). Representative enriched Gene Ontology (GO) terms within each cluster are indicated on the right (*P* value < 0.05). (**B**) Percentages (*x* axis) of genes differentially expressed in tissues (y axis) upon pretreatment with the PLA2G5 antibody in LPS-injected mice that match the genes regulated by LPS alone. BM, bone marrow; iLN, inguinal lymph node. (**C**) Heatmap of gene expression levels for genes related to splenic red pulp macrophage (rows) from whole-spleen mRNA profiles at 12 hours after sublethal LPS injection with or without PLA2G5 antibody pretreatment (columns). Values are normalized counts scaled by row (*n* = 3). (**D** and **E**) Images of Prussian blue staining for ferric iron on spleen histological sections from LPS-injected *Pla2g5*^+/+^ (top) or *Pla2g5*^–/–^ (bottom) mice (**D**). Insets in **D** match the magnified areas shown in **E**. Scale bars: 2 mm (**D**) and 200 μm (**E**). Images are representative of 2 independent experiments.

To investigate the mechanisms underlying the net, harmful effects of PLA2G5 during systemic inflammation, we measured changes in whole-tissue gene expression upon LPS injection across 12 organs (bone marrow, brain, colon, heart, inguinal lymph node, kidney, liver, lung, skin, small intestine, spleen, and thymus) from mice treated with the PLA2G5 antibody (Figure 4A, Supplemental Figure 7, A–C, and Supplemental Tables 1 and 2). PLA2G5 antibody treatment impacted 8.7% (387 of 4,443) of all genes regulated at 0.5 days after LPS injection across 7 out of the 12 organs profiled, and these effects on tissue states were most pronounced in the spleen, bone marrow, and lungs (Figure 4, A and B). Notably, by performing pathway enrichment analysis on these whole tissue expression profiles, we observed a link between PLA2G5 antibody treatment and changes in pathways related to splenic macrophages and iron homeostasis (Figure 4A and Supplemental Table 3). Since it is well established that splenic red pulp macrophages play a role in red blood cell recycling and iron homeostasis (35), it prompted us to explore the potential effects of PLA2G5 on these processes. By mining our whole-tissue gene expression profiles, we found that several genes linked to splenic red pulp macrophages, such as *Spic*, *Hmox1*, and *Vcam1* (36), were upregulated upon PLA2G5 antibody treatment in LPS-injected mice (Figure 4C), suggesting that PLA2G5 impacts the abundance of these splenic cells. Furthermore, the increase in splenic red pulp macrophages during systemic inflammation upon PLA2G5 antibody treatment was associated with a decrease in Prussian blue staining of ferric iron in the spleen (Figure 4, D and E).

### PLA2G5 leads to red blood cell lysis by hydrolyzing membrane phospholipids.

**Figure 5:**
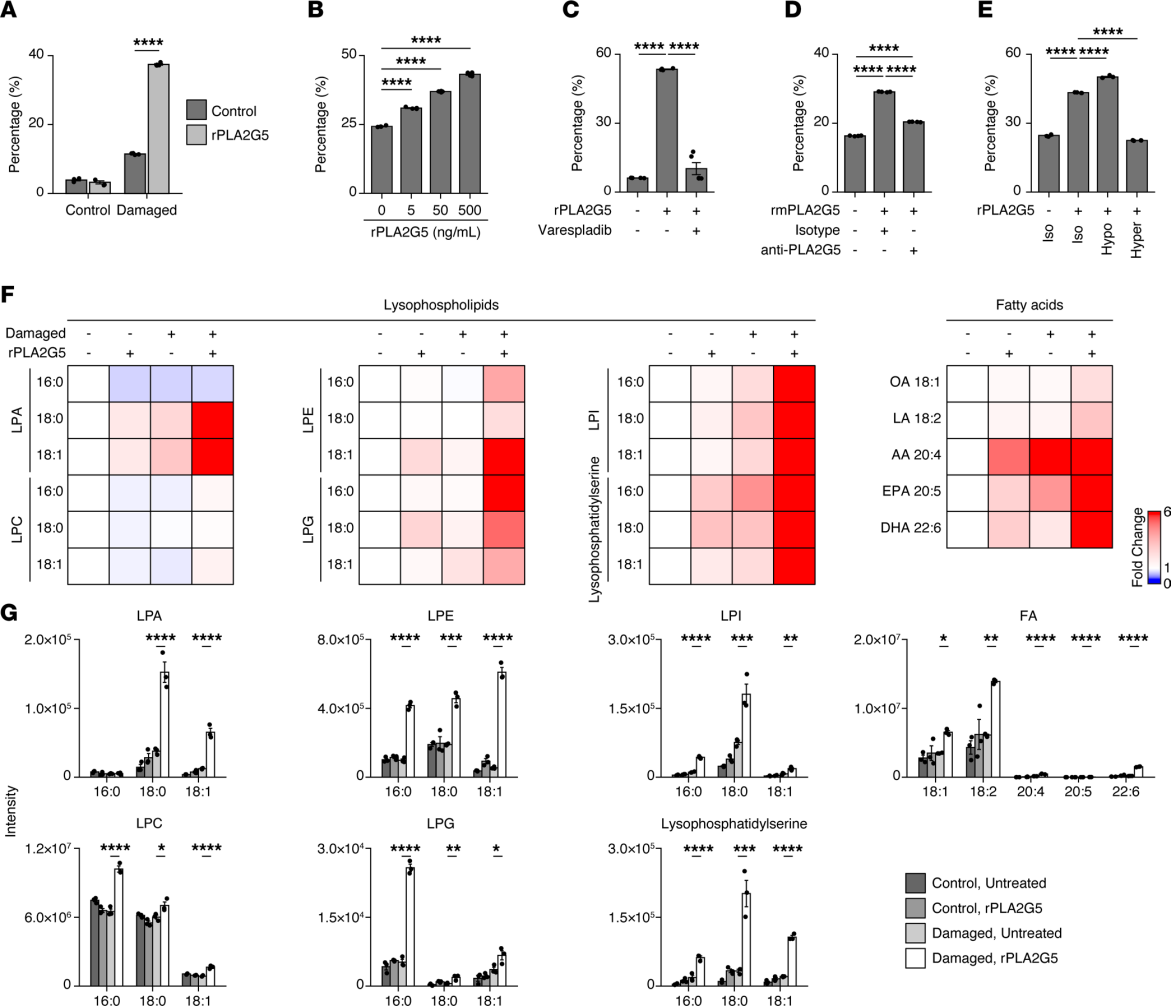
PLA2G5 lyses erythrocyte membrane phospholipids in vitro, leading to hemolysis. (**A**–**E**) In vitro hemolysis assay. Shown are percentages of cell-free oxyhemoglobin (relative to an equal volume of chemically lysed erythrocytes) in cell lysates from normal (control) or phosphatidylserine-exposing (PS-exposing; damaged) erythrocytes incubated with recombinant human PLA2G5 (rPLA2G5) or BSA for 1 hour at 37°C (**A**), PS-exposing erythrocytes incubated with indicated concentrations of rPLA2G5 for 1 hour at 37°C (**B**), PS-exposing erythrocytes incubated with rPLA2G5 in the presence or absence of the secreted phospholipase A2 inhibitor Varespladib or left untreated (control) (**C**), PS-exposing erythrocytes incubated with purified C-terminally His-tagged recombinant mouse PLA2G5 (rmPLA2G5) in the presence of PLA2G5 or isotype control IgG antibodies (**D**), or PS-exposing erythrocytes incubated with rPLA2G5 in isotonic, hypotonic, or hypertonic solution (**E**). Means ± SEM are shown (*n* = 4–5). Data are representative of 3 independent experiments. (**F** and **G**) Lipidomics profiling of normal or PS-exposing erythrocytes incubated with rPLA2G5 for 1 hour at 37°C. Shown are fold changes relative to normal erythrocytes (**F**) and mass spectrometry intensities (**G**) for indicated lipid metabolites. LPA, lysophosphatidic acid; LPC, lysophosphatidylcholine; LPE, lysophosphatidylethanolamine; LPG, lysophosphatidylglycerol; LPI, lysophosphatidylinositol; OA, oleic acid; LA, linoleic acid; AA, arachidonic acid; EPA, eicosapentaenoic acid; DHA, docosahexaenoic acid. Means ± SEM are shown (*n* = 3). *P < 0.05; **P < 0.01; ***P < 0.001; ****P < 0.0001 by 1-way ANOVA with Tukey-Kramer test.

We hypothesized that PLA2G5 impacted red blood cells because of the impact of PLA2G5 antibody treatment on splenic red pulp macrophages and iron stores. To test this hypothesis, we first asked if PLA2G5 could directly lyse red blood cells (RBCs) using an in vitro assay whereby recombinant human PLA2G5 protein (rPLA2G5) was mixed with mouse RBCs. rPLA2G5 led to increased RBC lysis in a dose-dependent manner in vitro upon treatment with Ca^2+^ and a calcium ionophore, which damages the plasma membrane of RBCs by disrupting the distribution of phospholipids (Figure 5, A and B). Furthermore, the hemolytic effects of rPLA2G5 were dependent on its phospholipase activity, as shown by adding varespladib, a broad inhibitor of secreted PLA_2_ enzymes, in our in vitro hemolytic assay (Figure 5C). Consistent with these findings, PLA2G5 antibody treatment significantly reduced hemolysis induced by recombinant mouse PLA2G5 (Figure 5D), indicating that the antibody can interfere with the hemolytic activity of recombinant mouse PLA2G5 in vitro. Next, we examined the effect of osmotic pressure on the hemolysis induced by rPLA2G5. The exposure of RBCs to hypertonic conditions inhibited hemolysis mediated by rPLA2G5, while the exposure to hypotonic conditions promoted hemolysis (Figure 5E), indicating that osmotic pressure affected the hemolytic activity of rPLA2G5.

To elucidate how PLA2G5 affected RBC membranes at the molecular level during hemolysis, we performed lipidomics on erythrocytes in the presence or absence of rPLA2G5 in vitro. We found that rPLA2G5 led to the release of lysophospholipids with saturation and lower degrees of unsaturation and fatty acids from RBC membranes in our in vitro hemolysis assay (Figure 5, F and G). Taken together, our data suggested that PLA2G5 hydrolyzed several phospholipids of damaged erythrocyte membranes, which led to red blood cell lysis through osmotic pressure changes in the plasma membrane.

### PLA2G5 mediates intravascular hemolysis in mice during systemic inflammation.

**Figure 6:**
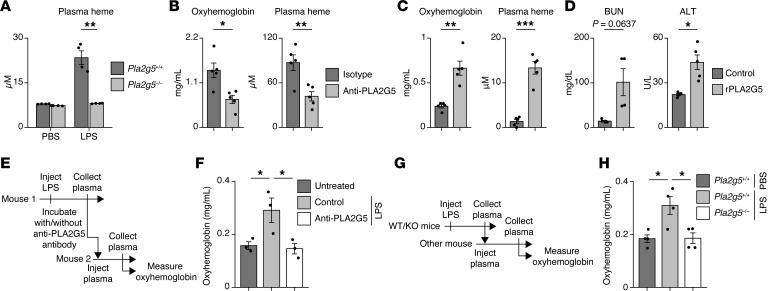
PLA2G5 acts as a hemolytic factor during systemic inflammation by mediating intravascular hemolysis. (**A**) Plasma heme concentrations in *Pla2g5*^+/+^ or *Pla2g5*^–/–^ mice 24 hours post-LPS or PBS (control) injections. Means ± SEM are shown (*n* = 4). (**B**) Plasma concentration of oxyhemoglobin and heme in mice 12 hours after lethal LPS injection and pretreated with the PLA2G5 antibody or isotype control. Means ± SEM are shown (*n* = 5). (**C** and **D**) Plasma concentration of oxyhemoglobin and heme (**C**) and serum levels of BUN and ALT (**D**) in mice injected with recombinant human PLA2G5 (rPLA2G5) or saline (control). Means ± SEM are shown (*n* = 4–5). (**E** and **F**) Plasma oxyhemoglobin levels in mice injected with plasma that were collected from LPS- or PBS (control)-injected mice and incubated in vitro with the PLA2G5 antibody for 4 hours at 4°C prior to transfer into naive mice. Means ± SEM are shown (*n* = 3). (**G** and **H**) Plasma oxyhemoglobin levels in mice injected with plasma collected from LPS-injected *Pla2g5*^+/+^ or *Pla2g5*^–/–^ mice or PBS-injected *Pla2g5*^+/+^ mice as control. Means ± SEM are shown (*n* = 4). Data are representative of 2–3 independent experiments (**A**–**D**, **F**, and **H**). *P < 0.05; **P < 0.01; ***P < 0.001 by 1-way ANOVA with Tukey-Kramer test (**A**, **F**, and **H**) or 2-tailed Student’s *t* test (**B**–**D**).

Next, we hypothesized that PLA2G5 impacted red blood cells in vivo during systemic inflammation. To test this idea, we measured the plasma levels of heme, a degradation product of cell-free hemoglobin, in *Pla2g5^+/+^* and *Pla2g5^–/–^* mice challenged with LPS. We found a significant decrease in the plasma levels of heme in *Pla2g5*-deficient mice compared with WT mice (Figure 6A). Moreover, PLA2G5 antibody treatment also inhibited the elevation of the plasma levels of oxyhemoglobin and heme in mice injected with a lethal dose of LPS (Figure 6B). Next, we asked if PLA2G5 would induce intravascular hemolysis in vivo by injecting rPLA2G5 in naive mice. We found that the systemic injection of rPLA2G5 in mice was sufficient to elevate the plasma levels of oxyhemoglobin and heme (Figure 6C) and the serum levels of BUN and ALT (Figure 6D) well above their physiological range of expression.

To test if PLA2G5 was present in the blood circulation and led to intravascular hemolysis in vivo during systemic inflammation, we performed plasma transfer experiments using donor plasma from WT and *Pla2g5^–/–^* mice treated with LPS. Donor plasma samples from WT mice injected with LPS were incubated with PLA2G5 or isotype control antibodies ex vivo prior to plasma transfer into naive mice. Donor plasma samples from *Pla2g5^–/–^* mice injected with LPS were directly injected into naive, WT mice. We found that plasma from LPS-injected WT mice led to an increase in hemolysis in naive mice, whereas plasma from PLA2G5 knockout or PLA2G5 antibody treatment did not (Figure 6, E–H).

### Serum levels of PLA2G5 in sepsis patients predict disease severity and mortality.

**Figure 7:**
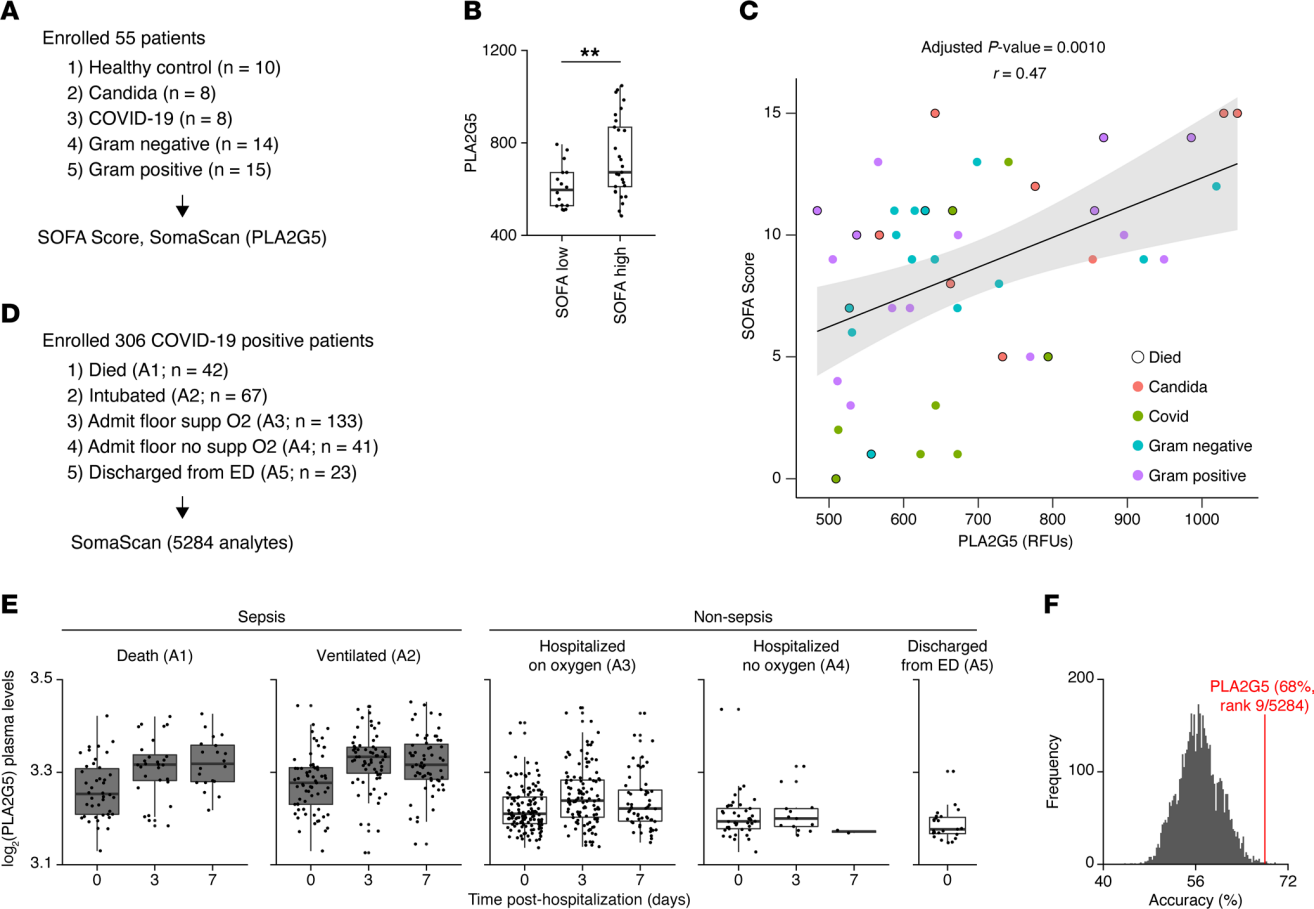
The serum level of PLA2G5 is elevated in humans with sepsis. (**A**) Schematic of study cohort. Serum samples were collected from patients from the University of Chicago hospital’s Medical Intensive Care Unit who matched the clinical definition of sepsis. Control samples were obtained from age-matched, healthy control individuals. (**B**) Box plot depicting the serum levels of PLA2G5 in septic patients with the sequential organ failure assessment (SOFA) scores of ≤ 7 versus ≥ 8. Box edges, interquartile range; middle line, median. **P < 0.01 by 2-tailed Student’s *t* test. (**C**) Correlation between the serum levels of PLA2G5 in patients with bacterial, fungal, or viral sepsis as indicated (colors) and SOFA score (data from **B**). Dots outlined in black indicate samples from patients who died from sepsis. (**D**) Schematic of study cohort in public blood proteomic data from patients with COVID-19. A1 is the most severe group and A5 is the least severe group. ED, Emergency Department. (**E**) Box plots depicting log_2_ relative fluorescence units of PLA2G5 level in plasma of patients with COVID-19 by severity over time (see Methods). Patients were classified by acuity levels based on the WHO Ordinal Outcomes Scale. Acuity levels were determined by their severity condition within 28 days after enrollment as follows: A1, death within 28 days; A2, intubation, mechanical ventilation; A3, hospitalized and requiring supplemental oxygen; A4, hospitalized without requiring supplemental oxygen; and A5, discharged directly from the Emergency Department without subsequently returning and requiring admission within 28 days. A1–A2 were classified as sepsis (dark grey) and A3–A5 as nonsepsis (white). Box edges, interquartile range; middle line, median. (**F**) Distribution of accuracy values from a decision tree classifier analysis using PLA2G5 plasma levels at day 0 from sepsis and nonsepsis patient groups (data from **E**).

**Figure 8:**
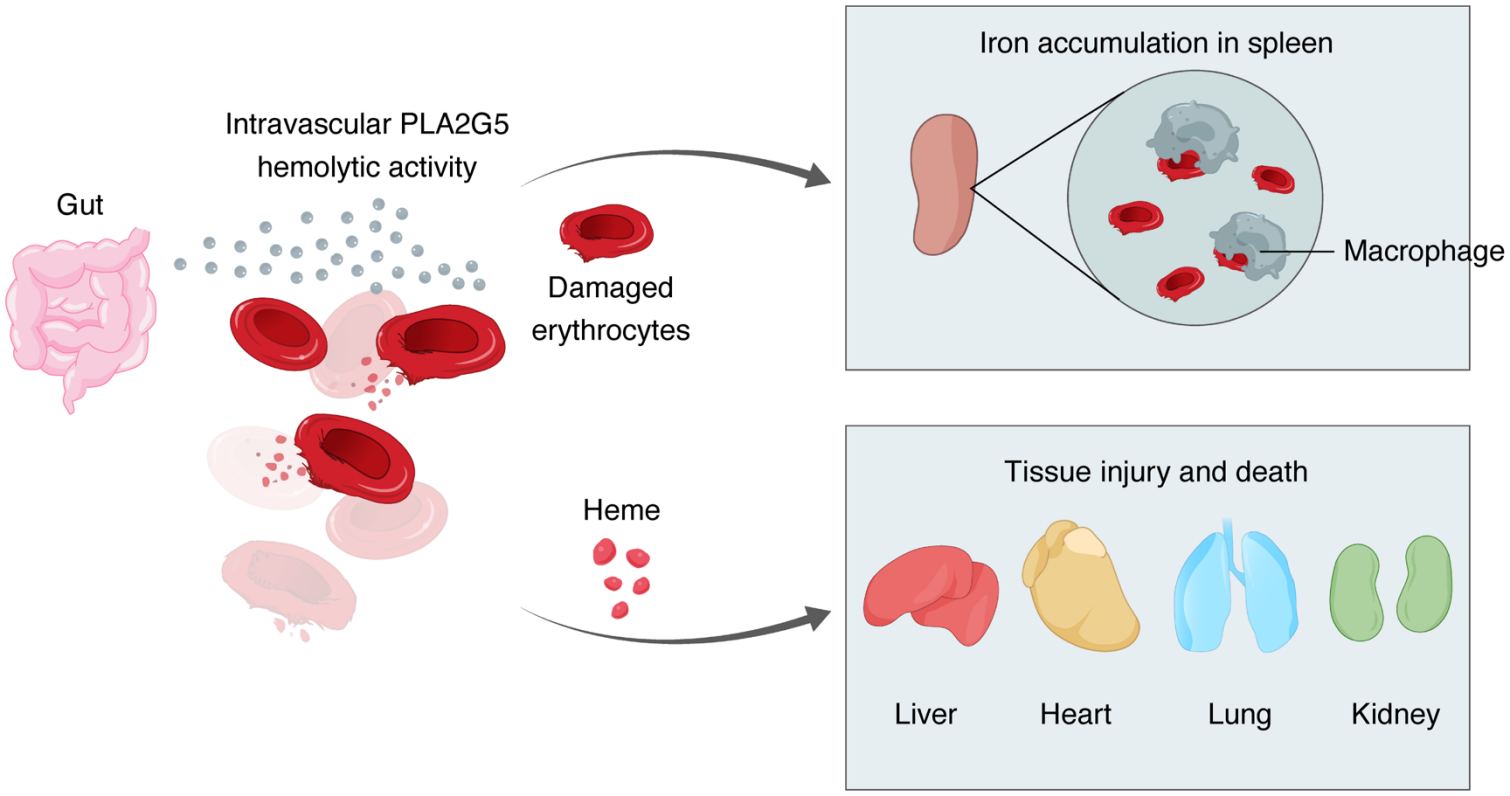
Schematic depicting the proposed model for the role of PLA2G5 in sepsis. PLA2G5 causes intravascular hemolysis through its lipolytic activity on select phospholipids of erythrocyte membranes. Damaged erythrocytes are captured by splenic macrophages, leading to iron accumulation in the spleen, and the degradation products from erythrocytes, such as heme, lead to toxic effects on tissues.

Lastly, we asked if PLA2G5 could be found in the blood circulation of patients with sepsis. We collected serum samples from patients from the University of Chicago hospital’s Medical Intensive Care Unit who matched the clinical definition of sepsis (1), and had either confirmed growth of bacteria including Gram-negative bacilli of order *Enterobacterales* (*n* = 14)*,* Gram-positive cocci of genus *Streptococcus* or *Staphylococcus aureus* in blood culture (*n* = 15), confirmed growth of *Candida spp*. in blood culture (*n* = 8), or a positive PCR test for SARS-CoV-2 (*n* = 8). We also obtained samples from age-matched, healthy control individuals (*n* = 10). Patients with multiple pathogens isolated from blood culture or with a mixed viral and bacterial infection were excluded. We measured the serum level of PLA2G5 using the SomaScan platform across our cohort of 55 individuals (Figure 7A, and Methods), and obtained the sequential organ failure assessment (SOFA) score used in the clinic to assess organ failure and mortality in patients who were critically ill (37). Septic patients with the SOFA scores of 8 or higher exhibited higher serum levels of PLA2G5 compared with those with scores of 7 or lower (Figure 7B). We also observed a positive correlation (adjusted *P* value = 0.0011) between the serum levels of PLA2G5 and the SOFA score (Figure 7C and Supplemental Table 4). To validate this observation in an independent cohort of patients, we used publicly available blood proteomic data from patients with COVID-19 (38) (Figure 7D). We found that the plasma levels of PLA2G5 were upregulated in patients with COVID-19–induced sepsis compared with milder, nonsepsis cases (Figure 7E). In addition, plasma PLA2G5 levels were predictive of patient survival with high accuracy compared with all other analytes measured in plasma (rank 9 in prediction accuracy for PLA2G5 out of a total of 5,284 analytes measured) (Figure 7F). Overall, our results support a model whereby the release of PLA2G5 into the systemic circulation during sepsis leads to intravascular hemolysis and a concurrent increase in the blood levels of heme, a known inducer of multiorgan injury (Figure 8).

## Discussion

The past 2 decades have broadened our understanding of the biology of the PLA_2_ family of phospholipase enzymes, as both secreted and cellular PLA_2_ enzymes have been linked to a wide array of homeostatic and pathological processes across tissues (14), prompting the search for inhibitors of these enzymes as potential therapies (39). Here, we uncovered an interorgan axis whereby bloodborne PLA2G5, a secreted phospholipase, led to hemolysis and multiorgan injury with lethal consequences for the host during sepsis. Treatment with the PLA2G5 antibody or genetic deletion decreased hemolysis and, concurrently, the burden of red blood cell death on splenic red pulp macrophages, iron homeostasis, and tissue damage due to the degradation products of erythrocytes. Mechanistically, PLA2G5 directly lysed red blood cells through its lipolytic activity. Our results suggest that PLA2G5 acts as a hemolytic factor with life-threatening consequences during sepsis by lysing host red blood cells in the bloodstream. Moreover, we found that the serum levels of PLA2G5 were increased in patients with sepsis, suggesting that this enzyme could be a candidate therapeutic target to decrease intravascular hemolysis in humans.

Another member of the secreted PLA_2_ family, PLA2G2A or sPLA2-IIA, is a sepsis biomarker that is thought to release arachidonic acid, the precursor of the eicosanoid biosynthetic pathways yielding pro- and antiinflammatory lipid species (14, 40, 41). Although PLA2G2A has been reported to hydrolyze phospholipids in vitro in erythrocyte-derived microvesicles (42) or activated or damaged erythrocyte membranes directly (43), the ability of PLA2G5 to hydrolyze cell membranes has been shown to be greater than that of PLA2G2A (44). Furthermore, intravascular hemolysis in sepsis has been shown to be independent of PLA2G2A in clinical studies (45). However, given that PLA2G2A is not expressed in C57BL/6J mice due to a frameshift mutation, further work is needed to test if PLA2G2A could play a role in intravascular hemolysis in mouse models in which the protein can be expressed and knocked out in the presence or absence of PLA2G5. The association between PLA2G5 protein levels in blood and disease severity across independent cohorts of patients with sepsis will require further investigation to determine the clinical implications of our findings.

It has been shown that PLA2G5 can act on the membranes of endothelial cells (19) and bacteria (14, 16). Thus, the expression of *Pla2g5* in mouse gut cells at a steady state might be indicative of a physiological role for PLA2G5 as a modulator of the microbiota, as shown for PLA2G2A (46, 47), although future work is needed to test this hypothesis. Interestingly, it has been reported that PLA2G5 is also expressed in cells of the human gastrointestinal tract, including in the stomach and colon lining (48, 49). However, it remains unknown whether PLA2G5 is induced in gut cells during human sepsis, as was observed in our mouse data. To address this gap, experimental models such as CLP are widely used to study sepsis. Their translational relevance must be interpreted with caution, because rodents and humans differ substantially in immune and physiological responses, particularly in their sensitivity to bacterial products such as LPS (50, 51). Accordingly, future studies evaluating these mechanisms in human tissues or patient cohorts will be important.

The gut has long been considered the “motor” of multiple organ dysfunction syndrome, with critical illness impacting the intestinal epithelium, immune system, and microbiome, which can propagate pathological systemic responses (52). Our findings extend this concept by suggesting that the gut can release mediators that act systemically to disrupt homeostasis, contributing to organ dysfunction in sepsis. Such gut-derived mediators may alter red blood cell homeostasis, immune cell distribution, and iron metabolism, thereby linking intestinal perturbations to distant organ injury and mortality. This perspective expands the traditional view of gut-driven pathology beyond barrier disruption and microbial translocation, highlighting the gut’s capacity to influence systemic physiology through secreted factors. While our data indicate that the gut is a significant source of PLA2G5 during sepsis, we cannot exclude contributions from other organs. Indeed, PLA2G5 is expressed in organs from control mice such as the spleen, kidney, and heart, but its localization and regulation in these tissues under homeostatic conditions may be compartmentalized or tightly controlled in ways that prevent its release into the circulation and potentially harmful effects. Future studies will be necessary to delineate the relative roles of different tissues in PLA2G5 production and their impact on systemic pathology.

Building on this perspective, the hemolytic activity of PLA2G5 in sepsis is reminiscent of the toxicity of sPLA_2_ present in snake venoms (53, 54). The increase in heme blood levels due to the hemolytic activity of PLA2G5 in septic blood and downstream multiorgan injury is consistent with previous work on the toxicity of heme on tissues during sepsis (11, 55). In addition, free hemoglobin has been reported to act in synergy with LPS, HMGB1, and multiple TLR agonists to enhance cytokine production (56–58). However, in our model, cytokine levels were not reduced in PLA2G5-deficient mice (Figure 3A), suggesting that the main pathological role of PLA2G5 during sepsis is intravascular hemolysis and heme-driven tissue injury rather than systemic cytokine induction. Although our work demonstrated that the dominant effect of PLA2G5 during sepsis is intravascular hemolysis, further work is needed to clarify if PLA2G5 might exert any other role across various tissues in sepsis. For example, the functional consequences, if any, of the observed decrease in *Pla2g5* mRNA levels in heart and spleen during sepsis remain to be investigated. Taken together, our data suggest that sepsis corrupts PLA2G5 —perhaps through the disruption of gut epithelia (59, 60) — into becoming a hemolytic factor lethal for the host.

## Methods

### Sex as a biological variable.

This study examined male and female animals, with similar findings reported for both sexes.

### Mice.

C57BL/6J mice (WT, stock 000664) were obtained from the Jackson Laboratories. *Pla2g5^–/–^* mice on a C57BL/6J genetic background were kindly provided by Steven Dudek (University of Illinois at Chicago) (23, 31). Experiments comparing *Pla2g5^+/+^* to *Pla2g5^–/–^* mice were performed using littermates or co-housed animals. Animals were housed in specific pathogen-free and BSL-2 conditions at The University of Chicago.

### Sepsis induction.

For LPS endotoxemia, mice were injected intraperitoneally with either lethal (10–15 mg/kg) or sublethal (3–5 mg/kg) doses of LPS derived from Escherichia coli O55:B5 (Sigma-Aldrich, L2880) diluted in PBS. Dosing was established for each lot of LPS by in vivo titration. Cecal ligation and puncture (CLP) was performed as described by others (26). Briefly, mice were anesthetized with isoflurane. A 1- to 2-cm midline laparotomy was performed, and the cecum was exposed. The cecum was ligated immediately below the ileocecal valve with 6-0 silk suture (Ethicon) and perforated twice with a 19-G needle. The cecum was tucked back into the peritoneum and gently squeezed to extrude a small amount of fecal content. The peritoneal wall was closed using absorbable suture. The skin was closed with surgical staples. To resuscitate animals, 1 mL of saline was injected subcutaneously. Mice were temporarily placed on a heating pad for recovery. Sham-operated mice underwent the same procedure except that the cecum was neither tied nor perforated. Cecal slurry injection was performed as described by others (61). Briefly, cecal contents were aseptically collected from healthy donor mice, filtered to remove large debris, mixed with an equal volume of 30% glycerol in phosphate-buffered saline, and stored at –80°C. The frozen cecal slurry was rapidly thawed by submerging the vial at 37°C, mixed thoroughly, and used immediately for injection with a 21-gauge needle.

### Antibody and drug treatment.

For PLA2G5 antibody treatment, C57BL/6J mice were injected intraperitoneally with 50 μg of PLA2G5 (clone MCL-3G1, Cayman Chemical, 160510), or mouse IgG1 isotype control antibodies (Clone MOPC-21, BioXCell, BE0083) in 100 μL of PBS 1 hour prior to LPS injection. For dexamethasone treatment, mice were injected intraperitoneally with 7 mg/kg of dexamethasone diluted in 100 μL of PBS 1 hour prior to LPS injection.

### Expression and purification of recombinant mouse Pla2g5.

For the production of mouse PLA2G5, a DNA fragment encoding mouse PLA2G5 with a C-terminal His tag was synthesized by Eurofins Genomics and cloned into the pcDNA3.1 (+) vector. The resulting expression vector was transfected into 293T cells (Riken BRC, RCB2202) using PEI Prime linear polyethylenimine (Sigma-Aldrich, 919012). Cells were lysed in 1% NP-40 lysis buffer (1% NP-40, 150 mM NaCl, 50 mM Tris-HCl, pH 8.0) supplemented with a cOmplete Mini EDTA-free Protease Inhibitor Cocktail (Roche, 11836170001). His-tagged PLA2G5 was purified using HisTrap FF columns (Cytiva, 17531901) and subsequently concentrated with Microsep Advance centrifugal filters with Omega membrane 3K (Cytiva, MCP003C41) according to the manufacturer’s instructions.

### Immunoprecipitation assay.

Immunoprecipitation (IP) was performed using Dynabeads Protein G (Thermo Fisher Scientific, 10003D) according to a standard protocol with minor modifications. Briefly, 100 μL of Dynabeads were washed 3 times with IP buffer (1% NP40, 150 mM NaCl, 50 mM Tris-HCl, pH 7.4) supplemented with EDTA-free protease inhibitor cocktail (Roche, 11836170001). Beads were incubated with 20 μg of PLA2G5 or isotype control IgG antibodies for 1 hour at 4°C with gentle rotation to allow antibody coupling. After coupling, beads were washed 3 times with IP buffer to remove unbound antibody. For each IP, purified C-terminally His-tagged recombinant mouse PLA2G5 was incubated with antibody-coated beads at 4°C for 6 hours with gentle rotation. Following incubation, beads were washed 3 times with ice-cold IP buffer. Washed beads were resuspended in SDS sample buffer and incubated for 10 minutes at 70°C to elute bound proteins. The eluted samples were analyzed by SDS-PAGE and Western blot using HRP-conjugated anti-His tag antibody (Jackson ImmunoResearch Laboratories, 300-035-240).

### Recombinant human PLA2G5 injections.

C57BL/6J mice were injected intravenously with 10 μg of recombinant human PLA2G5 (Cayman Chemical, 10009563).

### Blood analysis.

Mouse whole blood was harvested by cardiac puncture, and plasma and serum were isolated using lithium heparin-coated Microtainer blood collection tubes (BD, 365965) and Microtainer blood collection tubes (BD, 365978), respectively. For flow cytometric bead-based immunoassays, serum was diluted and processed using the LEGENDplex Mouse Macrophage/Microglia Panel (BioLegend, 740846) kit. Data were acquired on the NovoCyte flow cytometer (Acea Biosciences/Agilent) and analyzed using the LEGENDplex software v8 (BioLegend). To measure tissue injury marker levels in sera or plasma, samples were processed by In Vivo Animal Core, University of Michigan, with the following kits for BUN (BioAssay Systems, DIUR-100), ALT (Cayman Chemical, 700260), and troponin-I (Life Diagnostics, CTNI-1-HS) levels according to the manufacturer’s instructions, or with a Vet Axcel blood chemistry analyser (Alfa Wasserman). Total plasma heme was measured with the 3,3′,5,5′ tetramethylbenzidine (TMB) peroxidase assay and oxyhemoglobin with a Nanodrop One (Thermo Scientific) instrument.

### Tissue harvest.

Tissues were harvested, frozen and stored as previously described (62, 63). Mice were anesthetized with 2,2,2-tribromoethanol (250–500 mg/kg) and perfused transcardially with PBS containing 10 mM EDTA (to avoid signal contamination from blood in tissues). Prior to perfusion, blood was collected by cardiac puncture and stored on ice, and immediately after perfusion, tissues were placed in RNA-preserving solution (5.3 M ammonium sulfate, 25 mM sodium citrate, 20 mM EDTA) and kept at 4°C overnight prior to transfer at –80°C for storage. For each mouse, we harvested up to 12 tissues in total: lymph node (inguinal), flank skin, thymus, heart, lung, spleen, kidney, small intestine, colon, liver, brain, and bone marrow (BM). Small intestine and colon were cut longitudinally and washed extensively in PBS to completely remove feces contamination. Bone marrow cells were collected from femora and tibiae, stored overnight in RNA-preserving solution at 4°C, centrifuged at 5,000 *g* for 5 minutes at 4°C, and cell pellets were stored at –80°C.

### Whole-tissue RNA extraction.

Whole-tissue RNA extraction was performed as described previously (63). Briefly, tissues stored in RNA-preserving solution were thawed and transferred to 2 mL tubes containing 700–1500 μL (depending on tissue) of PureZOL (Bio-Rad, 7326890) or homemade Trizol-like solution (38% phenol, 0.8 M guanidine thiocyanate, 0.4 M ammonium thiocyanate, 0.1 M sodium acetate, 5% glycerol). Tissues were lysed by adding 2.8-mm ceramic beads (OMNI International, 19-646) and running 1–3 cycles of 5–45 seconds at 3500 rpm on the PowerLyzer 24 (QIAGEN). For liver, brain, and small intestine samples, tissues were lysed with 3-5 mL using M tubes (Miltenyi Biotec, 130-096-335) and running 1-4 cycles of the RNA_02.01 program on the gentleMACS Octo Dissociator (Miltenyi Biotec). Next, lysates were processed in deep 96-well plates (USA Scientific 1896-2000) by adding chloroform for phase separation by centrifugation, followed by precipitation of total RNA in the aqueous phase using magnetic beads coated with silane (Dynabeads MyOne Silane; ThermoFisher Scientific, 37002D), buffer RLT (QIAGEN, 79216), and ethanol. Genomic DNA contamination was removed by on-bead DNase I (ThermoFisher Scientific, AM2239) treatment at 37°C for 20 minutes. After washing steps with 80% ethanol, RNA was eluted from beads. This RNA extraction protocol was performed on the Bravo Automated Liquid Handling Platform (Agilent) (63). Sample concentrations were measured using a Nanodrop One (Thermo Scientific). RNA quality was confirmed using a Tapestation 4200 (Agilent Technologies).

### Whole-tissue RNA-seq.

For each tissue sample, full-length cDNA was synthesized in 20 μL final reaction volume containing the following: (a) 10 μL of 10 ng/μL RNA; (b) 1 μL containing 2 pmoles of a custom RT primer biotinylated in 5′ and containing sequences from 5′ to 3′ for the Illumina read 1 primer, a 6-bp sample barcode (up to 384), a 10-bp unique molecular identifier (UMI), and an anchored oligo(dT)_30_ for priming (64); and (c) 9 μL of RT mix containing 4 μL of 5X RT buffer, 1 μL of 10 mM dNTPs, 2 pmoles of template switching oligo (TSO), and 0.25 μL of Maxima H Minus Reverse Transcriptase (Thermo Scientific, EP0753). First, barcoded RT primers were added to RNA, which were then denatured at 72°C for 1 minute and snap cooled on ice. Second, the RT mix was added, and plates were incubated at 42°C for 120 minutes. For each library, double stranded cDNA from up to 384 samples were pooled using DNA Clean & Concentrator-5 columns (Zymo Research, D4013), and residual RT primers were removed using exonuclease I (New England Biolabs, M0293). Full-length cDNAs were amplified with 5 to 8 cycles of single-primer PCR using the Advantage 2 PCR Kit (clontech, 639206) and cleaned up using SPRIselect magnetic beads (Beckman Coulter, B23318). cDNA was quantified with a Qubit dsDNA High Sensitivity Assay Kit (ThermoFisher Scientific, 32851) and 50 ng of cDNA per pool of samples was tagmented using the Tagment DNA Enzyme I (Illumina, 20034197) and amplified using the NEBNext Ultra II Q5 Master Mix (New England BioLabs, M0544L). Libraries were gel purified using 2% E-Gel EX Agarose Gels (ThermoFisher Scientific, G402002), quantified with a Qubit dsDNA High Sensitivity Assay Kit (ThermoFisher Scientific, Q32851) and a Tapestation 4200 (Agilent Technologies), and sequenced on the NextSeq 550 platform (Illumina).

Sequencing read files were processed to generate UMI (unique molecular identifier) (65) count matrices using the python toolkit from the bcbio-nextgen project version 1.1.5 (https://bcbio-nextgen.readthedocs.io/en/latest/) (66). In brief, reads were aligned to the mouse mm10 transcriptome with RapMap (67). Quality control metrics were compiled with a combination of FastQC (http://bioinformatics.babraham.ac.uk/projects/fastqc/), Qualimap, MultiQC (https://github.com/ewels/MultiQC) (68, 69). Samples were demultiplexed using barcodes stored in Read 1 (first 6 bases) and raw UMI count matrices were computed using UMIs stored in Read 1 (bases 7 to 16) (https://github.com/vals/umis). Differential expression (DE) analysis was done using custom scripts in R (http://www.R-project.org). Raw count matrices were filtered to keep genes with at least 20 counts per million (cpm) or 5 UMIs in 2 samples and normalized across samples using the calcNormFactors function in edgeR (70). We identified genes with indicated Benjamini and Hochberg FDR adjusted *P* value by comparing treated tissues and matching control tissues using limma (71). Pathway enrichment analysis was done on differentially expressed genes from indicated k-means clusters using DAVID (72, 73). Heatmaps for RNA-seq data display the indicated numbers of transcripts and color intensities are determined by log2 fold change value for each heatmap. The rows of each heatmap were ordered by k-means clustering of log2 fold change values in R. All heatmaps were generated using ComplexHeatmap (https://github.com/jokergoo/ComplexHeatmap) and circlize (https://github.com/jokergoo/circlize) packages in R (74, 75).

### Histology.

Tissue processing, embedding, sectioning, and immunohistochemistry using H&E and Periodic Acid Schiff were performed by the Human Tissue Resource Center at the University of Chicago. To stain ferric iron in tissue sections, spleens were frozen in OCT using dry ice, sectioned (10 μm) using a cryostat (Leica CM1850), and stained with Perl’s Prussian blue (Sigma-Aldrich, P3289) and neutral red (Sigma-Aldrich, 72210). Section images were obtained using the Slideview VS200 Research Slide Scanner (Olympus).

### In vitro erythrocyte lysis assay.

Mouse whole blood was spun at 1,000*g*, the plasma and buffy coat were removed, and red blood cells were washed three times in Hepes-buffered saline. Subsequently, a portion of erythrocytes was incubated with 100 μM CaCl_2_ in Hepes-buffered saline for 3 minutes at 37°C. Calcium ionophore (Cayman Chemical, A23187) was added to a final concentration of 4 μM, and the cells were further incubated for 10 minutes at 37°C to induce membrane phospholipid (PS) scrambling. After that, 2 mM EDTA was added to stop the reaction. In the presence or absence of 100 nM of varespladib, hypertonic solution (100 mM NaCl), hypotonic solution (water-diluted Hepes-buffered saline), PLA2G5 antibody, or isotype control IgG, PS-exposing or untreated erythrocytes (10^9^ cells/mL) were incubated with 5, 50, or 500 ng/mL of recombinant human PLA2G5 (Cayman Chemical, 10009563), or purified C-terminally His-tagged recombinant mouse PLA2G5 for 1 hour at 37°C in Hepes-buffered saline with 2 mM of calcium for the activity of PLA2G5. Hemolysis was measured by determining the amount of oxyhemoglobin in the supernatant of erythrocyte suspensions after centrifugation at 4,000 *g*. The absorbance at 414 nm in the supernatant of erythrocyte suspensions was measured using NanoDrop (Thermo Scientific) and compared to that of a hemolyzed aliquot of the same erythrocyte suspension treated with Triton X-100.

### Lipidomics analysis of colon tissues.

The procedures for lipidomics analysis using high-performance liquid chromatography coupled with electrospray tandem mass spectrometry (LC-ESI-MS/MS) were described previously (76). Briefly, C57BL/6J were injected intraperitoneally with 50 μg of the PLA2G5 antibody in 100 μL of PBS 1 hour prior to LPS injection. Twelve hours after LPS injection, mice were anesthetized with 2,2,2-tribromoethanol (250–500 mg/kg) and perfused transcardially with PBS containing 10 mM EDTA. Immediately after perfusion, colon tissues were extensively washed in PBS and frozen by liquid nitrogen and kept at –80°C until the following procedures. Tissues were mechanically homogenized with the Precellys 24 homogenizer (Bertin Technologies, Montigny-le-Bretonneux, France) in methanol containing internal standards (500 pmol/sample of d4-labeled EPA, d5-labeled PGE2, LPC with a 17:0 fatty acyl chain (LPC17:0), and PC with two 14:0 fatty acyl chains (PE14:0-14:0)) and then incubated overnight at –20°C. For extraction of phospholipids and lysophospholipids, one-tenth of tissue lysates were added to 10 volumes of 20 mM Tris-HCl (pH 7.4) and were extracted using the method of Bligh and Dyer (77). For extraction of oxygenated fatty acid metabolites, nine-tenths of the tissue lysates were added to water (final methanol concentration of 10% (v/v)), and the lipids were extracted using an Oasis HLB cartridge (Waters, Milford, MA, USA). The samples were applied to a Kinetex C18 column (Kinetex C18, 2.1 x 150 mm, 1.7 μm particle; Phenomenex, Inc.) connected with ESI-MS/MS on a liquid chromatography (NexeraX2 system; Shimadzu Co.) coupled with a 4000Q-TRAP quadrupole-linear ion trap hybrid mass spectrometer (AB Sciex). For analyses of free fatty acids (FFAs), lysophospholipids (LPLs) and phospholipids, the samples were applied to the column and separated by a step gradient with mobile phase A (acetonitrile/methanol/water =1/1/1 (v/v/v) containing 5 mM phosphoric acid and 1 mM ammonium formate) and mobile phase B (2-propanol containing 5 μM phosphoric acid and 1 mM ammonium formate) at a flow rate of 0.2 mL/min at 50°C. For analyses of oxygenated fatty acid metabolites, the samples were applied to the column and separated using a step gradient including mobile phase C (water containing 0.1 % acetic acid) and mobile phase D (acetonitrile/methanol = 4/1 (v/v)) at a flow rate of 0.2 mL/ min at 45°C. Identification of phospholipids, LPLs, FFAs, and oxygenated PUFAs (polyunsaturated fatty acids) metabolites was conducted by multiple reaction monitoring (MRM) transition, and quantification was performed based on the peak area of the MRM transition and the calibration curve obtained with an authentic standard for each compound (78).

### Lipidomics analysis of erythrocytes.

The procedures for lipidomics analysis using high-performance liquid chromatography coupled with electrospray tandem mass spectrometry (LC-ESI-MS/MS) were described above. Mouse whole blood was spun at 1,000*g*, the plasma and buffy coat were removed, and red blood cells were washed three times in Hepes-buffered saline. Subsequently, a portion of erythrocytes was incubated with 100 μM CaCl_2_ in Hepes-buffered saline for 3 minutes at 37°C. Calcium ionophore (Cayman Chemical, A23187) was added to a final concentration of 4 μM, and the cells were further incubated for 10 minutes at 37°C to induce membrane phospholipid (PS) scrambling. After that, 2 mM EDTA was added to stop the reaction. PS-exposing or untreated erythrocytes (10^9^ cells/mL) were incubated with or without 100 ng/mL of recombinant human PLA2G5 (Cayman Chemical, 10009563) for 1 hour at 37°C in Hepes-buffered saline with 2 mM of calcium for the activity of PLA2G5. The samples were kept at –80°C until the following procedures.

### Plasma transfer.

Whole blood was harvested by cardiac puncture from donor *Pla2g5^+/+^* or *Pla2g5^–/–^* mice injected with LPS 12 hours prior to bleeding animals. Plasma was isolated using lithium heparin coated Vacutainer tubes (BD) and incubated with or without the PLA2G5 antibody for 4 hours at 4°C. Plasma treated with the PLA2G5 antibody was transferred intravenously (100 μL) to recipient C57BL/6J mice. Plasma oxyhemoglobin in recipient mice was measured 16 hours after plasma transfer.

### Whole-mouse spatial transcriptomics.

Spatial transcriptomics was performed as described previously by us (25, 79). Mice injected with LPS or left untreated as control were euthanized with CO_2_, frozen in a dry ice-hexane bath after removing all body hair and teeth, and stored at –80°C until use. Frozen mice were embedded in a cryo-embedding medium and sectioned (10-μm thickness) using a Leica CM3600-XP cryomacrotome. Resulting whole-mouse sections were transferred onto custom, large-format microarrays for spatial transcriptomics (ST) (30-μm spot diameter with 36.65 μm center-to-center distance between spots). After transfer, sections were fixed in methanol, stained with hematoxylin and eosin, and imaged on an Olympus VS2000 slide scanner (20X magnification). Sections were permeabilized (1% pepsin), incubated for in-tissue reverse transcription, and treated with Proteinase K for tissue removal. Resulting full-length, single-stranded cDNAs were denatured and retrieved from the array using KOH and purified by column clean up (Zymo Research). cDNA was processed for single-primer PCR amplification followed by sequencing library construction using tagmentation (Nextera DNA Library Prep Kit) and final PCR amplification. Resulting libraries were sequenced on the NovaSeq 6000 (Illumina) and sequencing data was pre-processed using STAR/STARsolo 2.7.10a (80) (https://github.com/alexdobin/STAR/blob/master/docs/STARsolo.md) for read alignment using the GRCm39 mouse reference genome, spatial barcode demultiplexing, and unique molecular identifier (UMI) counting. Resulting ST data was normalized, processed for differential expression analysis, and visualized using custom Python 3.8.5 (http://www.python.org) scripts and existing packages, including Scanpy (81), scikit-image (82), and Seaborn (83, 84). Cell type deconvolution for each ST spot was done using the CARD package (28).

### Sepsis patient cohort serum PLA2G5 analysis.

Serum samples from healthy and sepsis-patient samples were analyzed using the SomaScan Platform for proteomic profiling which relies on single-stranded DNA aptamers specific to unique human protein targets. The dataset consisted of abundance measurements of 1,512 proteins in 45 patients with sepsis and 10 healthy controls. Of the 45 sepsis cases, 8 were attributed to Candida infection, 8 to SARS-CoV-2 infection, 14 to Gram-Negative bacterial infection, and 15 to Gram-Positive bacterial infection (Supplemental Table 4). The correlation of serum protein levels and clinical Sequential Organ Failure Assessment (SOFA) scores was estimated by fitting the protein abundance measurements in linear models (Empiric Bayes approach) using the limma R package (71), including etiological agents as covariates. The Benjamini-Hochberg approach was used for multiple testing correction. Correlations with adjusted *P*-val < 0.05 were considered significant. The correlation coefficient of the model fit was calculated using the glm package in R.

### Public RNA-seq data.

To assess the expression pattern of *Pla2g5* across the body, we obtained single-cell RNA-seq data from the Tabula Muris Senis website (https://figshare.com/projects/Tabula_Muris_Senis/64982) and used the package TabulaMurisSenisData (github.com/fmicompbio/TabulaMurisSenisData) for data visualization (29, 85). In addition, we used bulk RNA-seq data (GSE224146) previously reported by us (25) to assess the expression pattern of *Pla2g5* across organs. We also reanalyzed spatial transcriptomics sequencing data and H&E-stained images from the same previously reported dataset (GSE224146), which was originally generated and published in our previous study (25).

### Public proteomic and clinical index data.

To assess the protein level of PLA2G5 in plasma from COVID-19 patients with sepsis, we obtained proteomic and clinical index data from Mendeley Data (https://data.mendeley.com/datasets/nf853r8xsj/2) (38).

### Decision-tree analysis.

We used the Python package SciPy package scikit-learn for decision-tree analysis (86). We trained the decision-tree algorithm DecisionTreeClassifier. For cross-validation, we first separated the data into training and test sets using cross_validate with cv=5, trained the decision-tree algorithm DecisionTreeClassifier, and then classified the test data set.

### Structural illustrations and comparison.

PDB files (AF-P39877-F1-v4 for human PLA2G5, and AF-P97391-F1-v4 for mouse PLA2G5) were downloaded from AlphaFold Protein Structure Database (https://alphafold.ebi.ac.uk/). DALI server (http://ekhidna2.biocenter.helsinki.fi/dali/) was used for structural comparison (30). All molecular graphics figures were prepared with CueMol2 (Ishitani; https://cuemol.github.io/cuemol2_docs/en/).

### Statistics.

The statistical significance was determined using limma, 2-tailed Student’s *t* test, or 1-way ANOVA with Tukey-Kramer test. Survival analysis was performed using the log-rank test. *P* values are denoted by *P < 0.05, **P < 0.01, ***P < 0.001, ****P < 0.0001.

### Study approval.

All animal experiments were performed in accordance with the US National Institutes of Health Guide for the Care and Use of Laboratory Animals and approved by The University of Chicago Institutional Animal Care and Use Committee. Serum samples were collected from patients from the University of Chicago hospital’s Medical Intensive Care Unit. All studies involving humans were approved by the Institutional Review Board at the University of Chicago, and informed written consent was obtained from all participants prior to the commencement of the study.

### Data availability.

The sequencing (whole-tissue and spatial RNA-seq) and mass spectrometry (lipidomics) datasets generated during this study have been respectively deposited in the Gene Expression Omnibus and National Metabolomics Data Repository under accession numbers GSE253060 and ST002455, respectively. Values for all data points in graphs are reported in the Supporting Data Values file. All scripts and preprocessed datasets are publicly available at the following repository: https://doi.org/10.5281/zenodo.17774346

## Author contributions

MT and NC conceptualized and designed methodology. The investigation was performed by MT with assistance from GR, MP, and TU. YM, YN, YT, and MM performed the lipidomics. KSW and AC curated the sepsis patient samples. DC and NC conducted the spatial transcriptomics. MT, FH, and NC performed the data analysis. TS, TK, SMD, and NC provided resources. MT and NC wrote the original draft. All authors contributed to the review and editing of the manuscript. MT and NC acquired funding. NC supervised the study.

## Conflict of interest

The authors have declared that no conflict of interest exists.

## Funding support

This work is the result of NIH funding and is subject to the NIH Public Access Policy. Through acceptance of this federal funding, the NIH has been given a right to make the work publicly available in PubMed Central.

- Astellas Foundation for Research on Metabolic Disorders (MT).
- Senri Life Science Foundation (MT).
- Chugai Foundation for Innovative Drug Discovery Science: C-FINDs (MT).
- Takeda Science Foundation (MT).
- Grant-in-Aid for Scientific Research (B) 25K03064 from the Japan Society for the Promotion of Science (MT).
- AMED-CREST JP22gm1210013 from the Japan Agency for Medical Research and Development (MM).
- Grant-in-Aid for Scientific Research (S) JP20H05691 from the Japan Society for the Promotion of Science (MM).
- NIH grants DP2-AI145100 and U01-AI160418 (NC).
- CZI grant DAF2020-217464 and grant DOI https://doi.org/10.37921/767230ofotux from the Chan Zuckerberg Initiative DAF, an advised fund of Silicon Valley Community Foundation (funder DOI 10.13039/100014989) (NC).
- The University of Chicago Medicine Comprehensive Cancer Center (UCCCC) Janet D. Rowley Discovery Fund (NC).
- The University of Chicago Center for Interdisciplinary Study of Inflammatory Intestinal Disorders (C-IID) (P30 DK42086) (NC).
- The Chicago Immunoengineering Innovation Center (NC).
- The Pritzker School of Molecular Engineering at the University of Chicago (NC).

## Supplementary Material

Supplemental data

Unedited blot and gel images

Supplemental tables 1-4

Supporting data values

## Version 1 05/01/2026

Electronic publication
