# Supplementary material for: Secreted phospholipase PLA2G5 acts as a hemolytic factor in sepsis
Source: J Clin Invest. 2026 May 1;136(9):e195001. doi: 10.1172/JCI195001 (PMC13132393; doi:10.1172/JCI195001)
Supplement: Supplemental data [file jci-136-195001-s021.pdf]

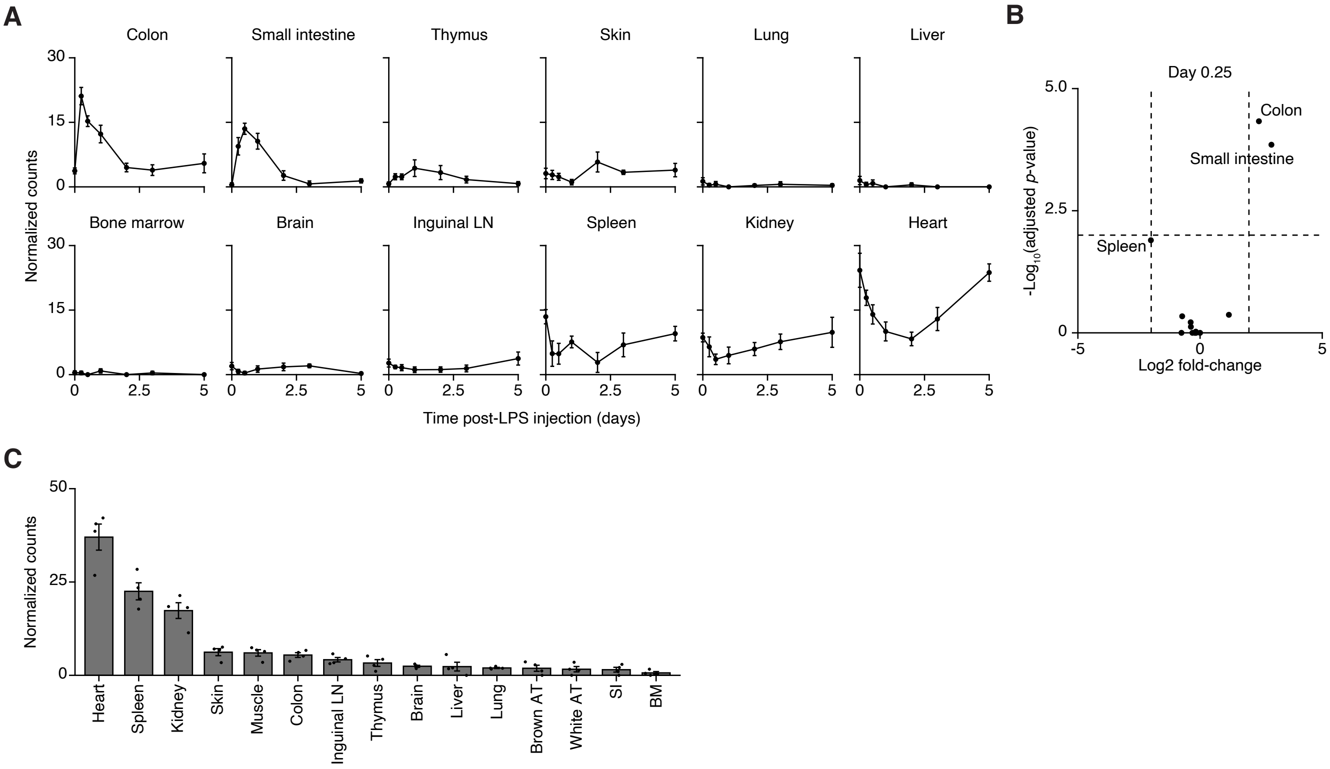

Supplemental Figure 1. Transcriptional regulation of *Pla2g5* across tissues.
(**A**) Normalized counts for *Pla2g5* from indicated organs during systemic inflammation. LN, lymph node. Means ± SEM are shown (n = 3-4).
(**B**) Volcano plot for *Pla2g5* expression at 6 hours post-LPS injection across all 12 organs (circles) from **A**.
(**C**) Basal whole-tissue gene expression of *Pla2g5* mRNAs across 15 tissues. Shown are normalized counts for *Pla2g5* from indicated organs in naive mice. LN, lymph node; AT, adipose tissue; SI, small intestine; BM, bone marrow. Data are shown as means ± SEM (n = 4).

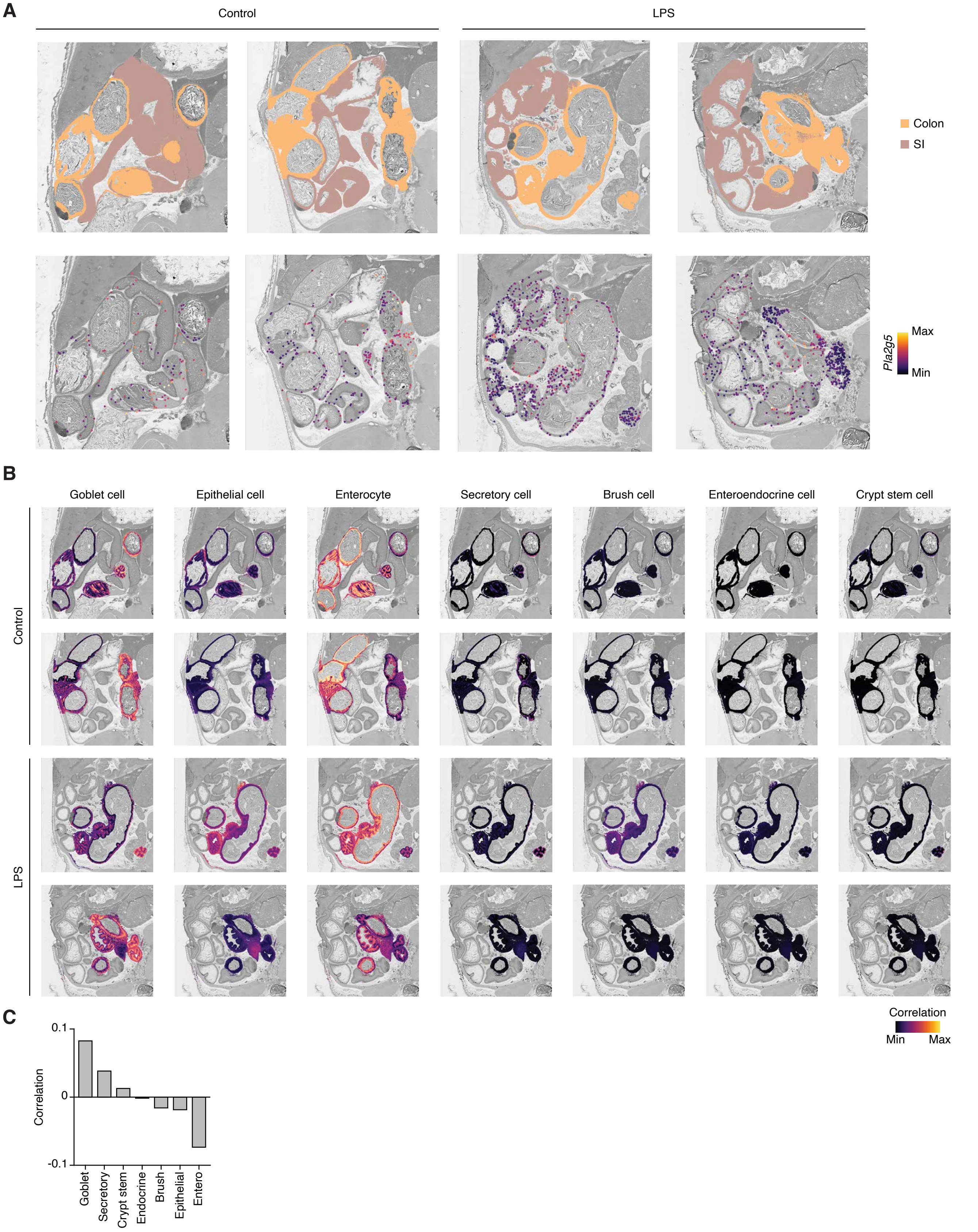

Supplemental Figure 2. Spatial transcriptomics analysis of *Pla2g5* expression in the gut.
(**A**) Whole-mouse spatial transcriptomics (ST) data of tissue clusters generated by unsupervised clustering and *Pla2g5* mRNA levels from control and LPS conditions (columns) were magnified to only show colon (brown) and small intestine (SI, yellow) tissues (top) and *Pla2g5* normalized expression (bottom) overlaid on a greyscale H&E image. The images shown in this figure are magnified views of the data presented in Figure 1A.
(**B**) Cell type deconvolution analysis of each ST spot data for indicated colon cell types (top) across control and LPS conditions (rows).
(**C**) Correlation between *Pla2g5* expression and cell type deconvolution predictions across each ST array spot under the colon tissue. Shown are goblet cells, secretory cells, enteroendocrine cells, brush cells, epithelial cells, and crypt stem cells and enterocytes.

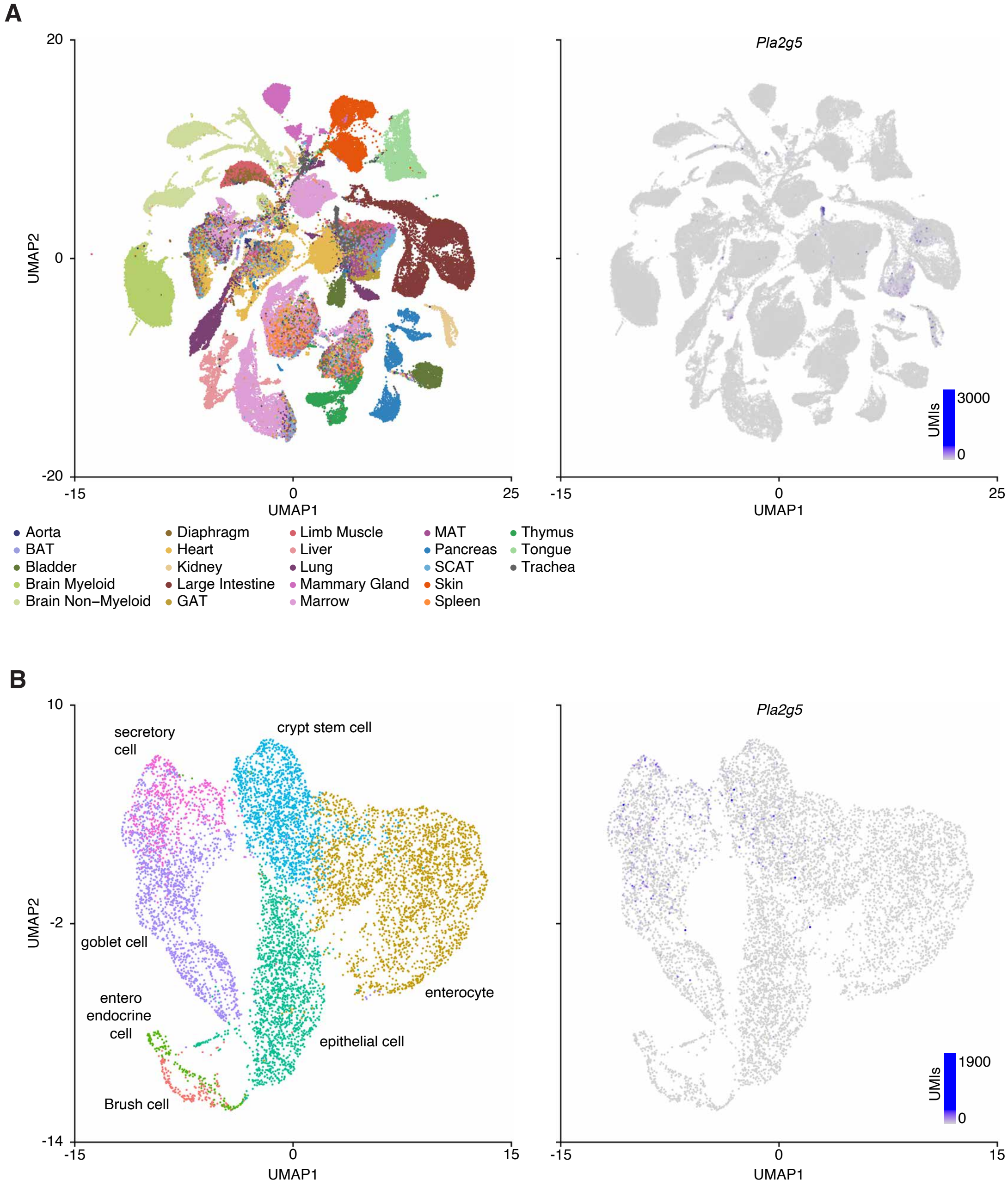

Supplemental Figure 3. *Pla2g5* expression in public single-cell RNA-seq data across mouse tissues.
(**A-B**) Single-cell expression of *Pla2g5* mRNAs across mouse tissues from the public database Tabula Muris Senis. Shown are uniform manifold approximation and projection (UMAP) plots of *Pla2g5* mRNA counts (right panels; **A-B**) across indicated mouse organs (left panel; **A**) or large intestine cell types (left panel; **B**). BAT, brown adipose tissue; GAT, gonadal adipose tissue; MAT, mesenteric adipose tissue; SCAT, subcutaneous adipose tissue.

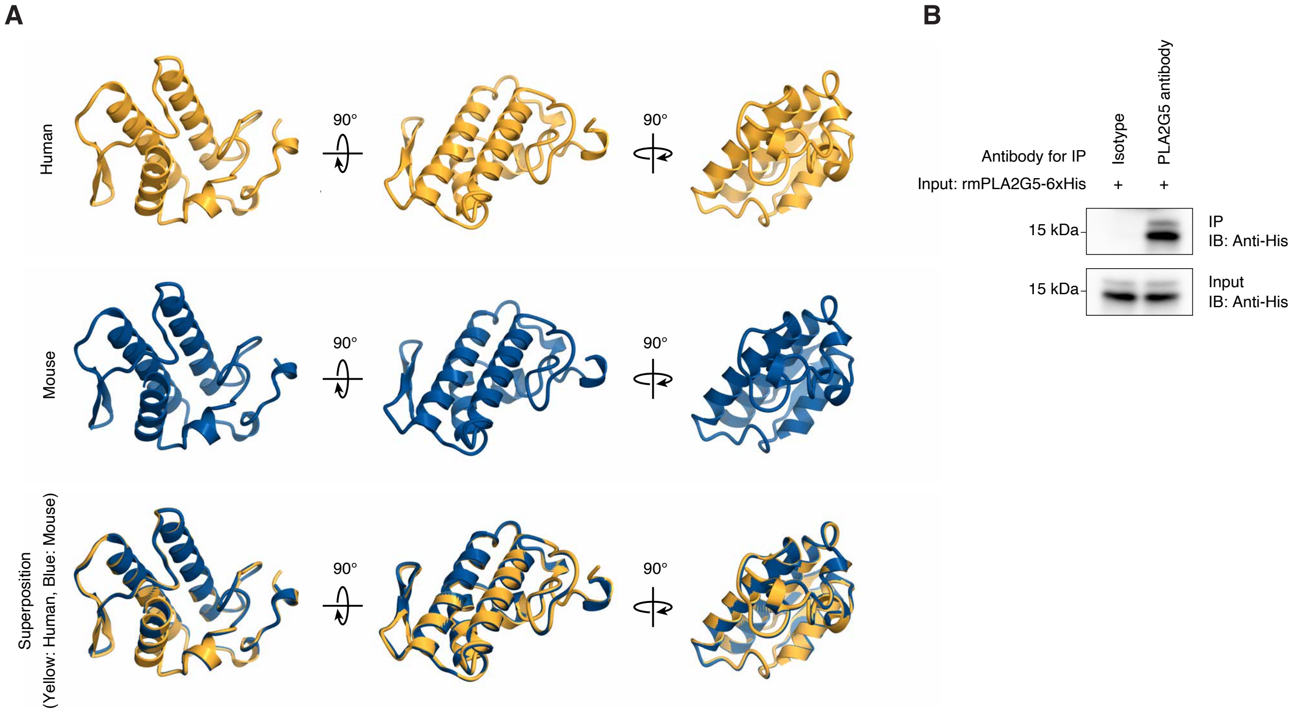

Supplemental Figure 4. MCL-3G1 interacts with mouse PLA2G5.
(**A**) Human PLA2G5 (yellow) superimposed onto mouse PLA2G5 (blue) from three angles. The signal peptide was removed from both proteins prior to generating the structural models.
(**B**) Immunoprecipitation assay of purified C-terminally His-tagged recombinant mouse PLA2G5 (rmPLA2G5) using the PLA2G5 antibody (clone MCL-3G1).

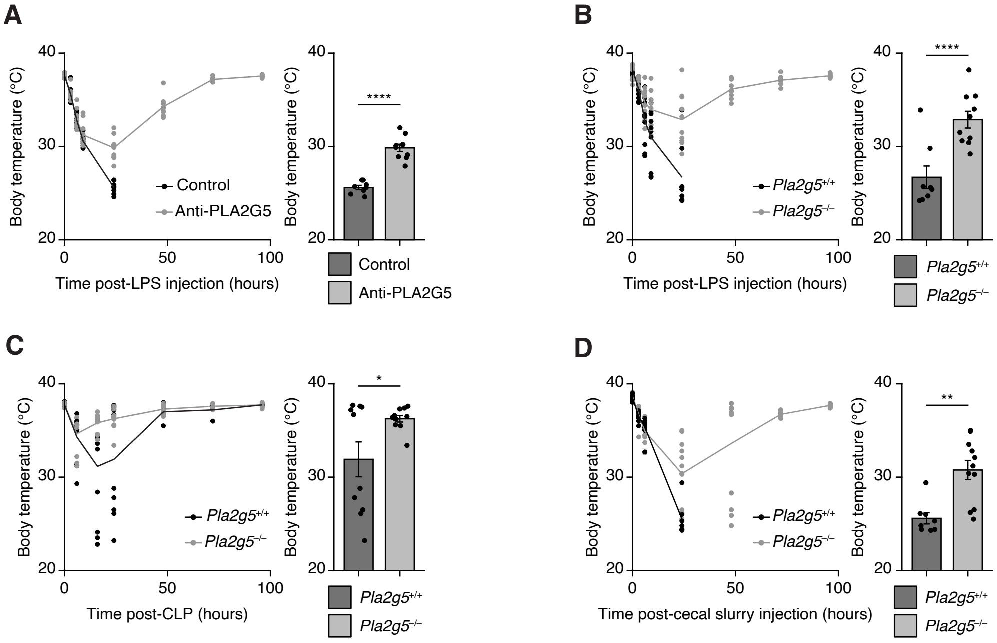

Supplemental Figure 5. PLA2G5 perturbation prevents body temperature loss in sepsis.

(**A-D**) Measurements of rectal temperature in mice upon LPS-induced endotoxemia (**A-B**), cecal ligation and puncture sepsis (CLP sepsis, **C**) or cecal slurry-induced sepsis (**D**) using the PLA2G5 antibody (**A**) or indicated mouse genotypes (**B-D**).

Data are representative of three independent experiments. *, $P < 0.05$; **, $P < 0.01$; ****, $P < 0.0001$ by two-tailed Student's *t* test (**A-D**).

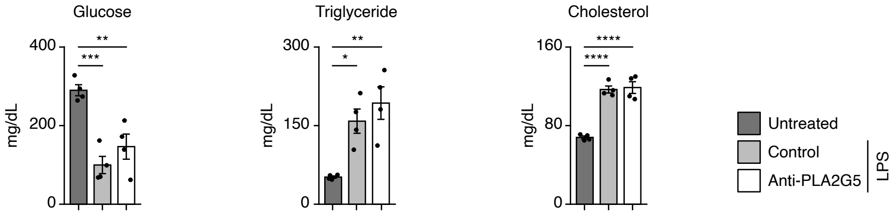

Supplemental Figure 6. Impact of PLA2G5 on the plasma levels of glucose, triglyceride, and cholesterol during systemic inflammation.

Serum levels of the indicated metabolites before (untreated) and 24 hours post-injection with a sublethal LPS dose in the presence or absence of PLA2G5 antibody treatment.

Data are representative of two independent experiments and shown as means ± SEM (n = 4). *, $P < 0.05$; **, $P < 0.01$; ***, $P < 0.001$; and ****, $P < 0.0001$ by one-way ANOVA with Tukey-Kramer test.

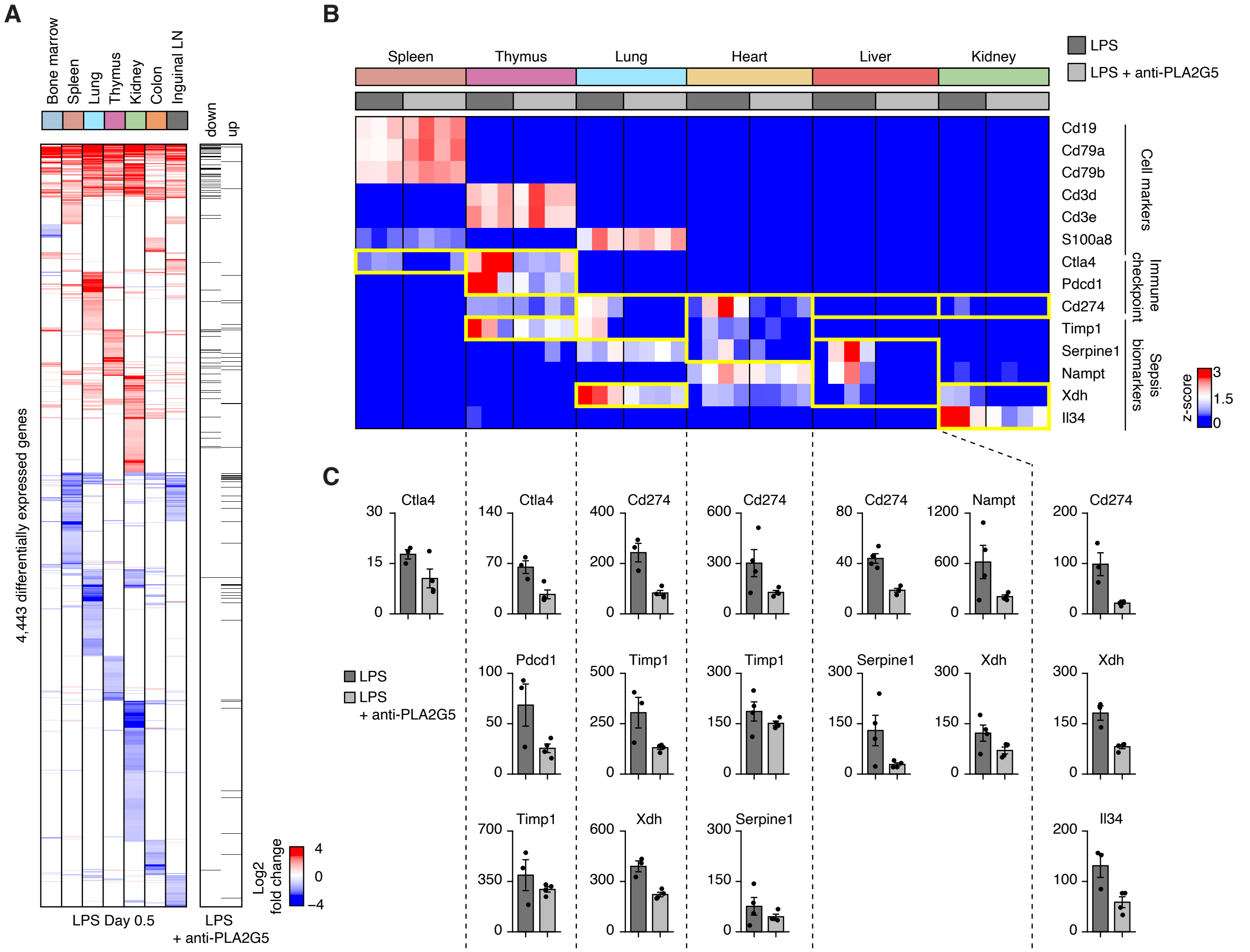

Supplemental Figure 7. PLA2G5 antibody treatment impacts multi-tissue gene expression during systemic inflammation.
(**A**) Heatmap of differentially expressed genes (rows) from whole-tissue mRNA profiles ordered by k-means clustering and organ types (top, colors) at 12 hours after sublethal LPS injection (left). Values are log2 fold-changes relative to matching organs from untreated controls for wild-type (FDR-adjusted p-value < 0.01, absolute fold change > 2; n = 4). Genes up- or down-regulated in at least one of the 7 tissues at 12 hours post-sublethal LPS injection in the presence of PLA2G5 antibodies (FDR-adjusted p-value < 0.01; n = 3-4) are indicated in black (right).
(**B**) Heatmap of gene expression levels for indicated cell-type markers and sepsis biomarker genes (rows) from mRNA profiles at 12 hours after sublethal LPS injection in the presence (light grey) or absence (dark grey) of PLA2G5 antibodies. Values are normalized counts scaled by row. All genes shown were significantly downregulated by PLA2G5 antibody treatment in LPS-treated mice (FDR-adjusted p-value < 0.01; n = 3-4).
(**C**) Bar plots of normalized counts for indicated genes and organs from panel **B**. Data are shown as means ± SEM (n = 3-4).
