# Supplementary figures and images for: Secreted phospholipase PLA2G5 acts as a hemolytic factor in sepsis
Source: J Clin Invest. 2026 May 1;136(9):e195001. doi: 10.1172/JCI195001 (PMC13132393; doi:10.1172/JCI195001)

Uncropped blot for Supplemental Figure 4B

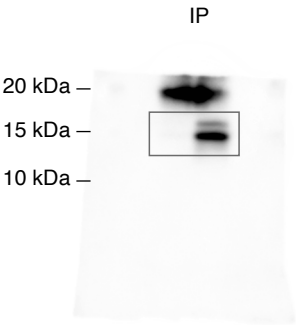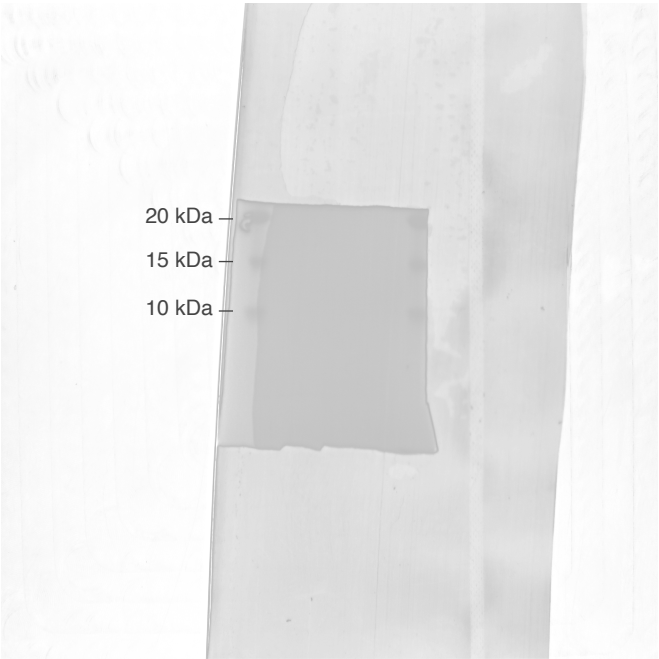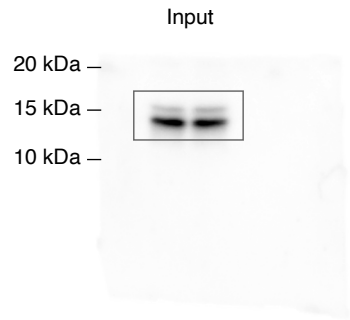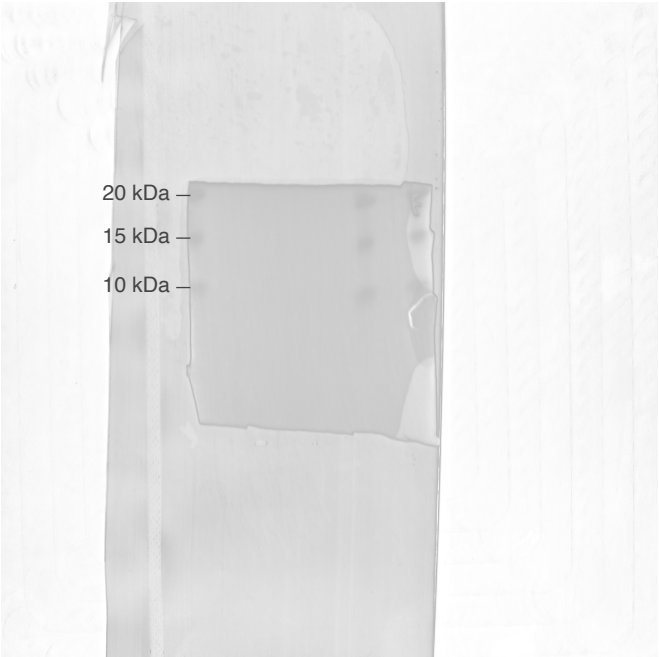

Supplement: Unedited blot and gel images [file jci-136-195001-s022.pdf]
